# Prevotellaceae produces butyrate to alleviate PD-1/PD-L1 inhibitor-related cardiotoxicity via PPARα-CYP4X1 axis in colonic macrophages

**DOI:** 10.1186/s13046-021-02201-4

**Published:** 2022-01-03

**Authors:** Yaxin Chen, Yanzhuo Liu, Yang Wang, Xuewei Chen, Chenlong Wang, Xuehan Chen, Xi Yuan, Lilong Liu, Jing Yang, Xiaoyang Zhou

**Affiliations:** 1 Department of Cardiology, Renmin Hospital of Wuhan University, Wuhan, 430060 China; 2 Department of Pharmacy, Renmin Hospital of Wuhan University, Wuhan, 430060 China; 3 Department of Pharmacology and Hubei Province Key Laboratory of Allergy and Immune-related Diseases, School of Basic Medical Sciences, Wuhan University, Wuhan, 430071 China; 4 Department of Laboratory Medicine, Zhongnan Hospital of Wuhan University, Wuhan, 430071 China; 5 Department of Pharmacy, Zhongnan Hospital of Wuhan University, Wuhan, 430071 China

**Keywords:** PD-1/PD-L1 inhibitors, Cardiotoxicity, Gut microbiota, The colonic macrophage, CYP4X1

## Abstract

**Background:**

Immune checkpoint inhibitor-related cardiotoxicity is one of the most lethal adverse effects, and thus, the identification of underlying mechanisms for developing strategies to overcome it has clinical importance. This study aimed to investigate whether microbiota-host interactions contribute to PD-1/PD-L1 inhibitor-related cardiotoxicity.

**Methods:**

A mouse model of immune checkpoint inhibitor-related cardiotoxicity was constructed by PD-1/PD-L1 inhibitor BMS-1 (5 and 10 mg/kg), and cardiomyocyte apoptosis and cardiotoxicity were determined by hematoxylin and eosin, Masson’s trichome and TUNEL assays. 16S rRNA sequencing was used to define the gut microbiota composition. Gut microbiota metabolites short-chain fatty acids (SCFAs) were determined by HPLC. The serum levels of myocardial enzymes (creatine kinase, aspartate transaminase, creatine kinase-MB and lactate dehydrogenase) and the production of M1 factors (TNF-α and IL-1β) were measured by ELISA. The colonic macrophage phenotype was measured by mmunofluorescence and qPCR. The expression of Claudin-1, Occludin, ZO-1 and p-p65 was measured by western blot. The gene expression of peroxisome proliferator-activated receptor α (PPARα) and cytochrome P450 (CYP) 4X1 was determined using qPCR. Statistical analyses were performed using Student’s t-test for two-group comparisons, and one-way ANOVA followed by Student–Newman–Keul test for multiple-group comparisons.

**Results:**

We observed intestinal barrier injury and gut microbiota dysbiosis characterized by *Prevotellaceae* and *Rikenellaceae* genus depletion and *Escherichia-Shigella* and *Ruminococcaceae* genus enrichment, accompanied by low butyrate production and M1-like polarization of colonic macrophages in BMS-1 (5 and 10 mg/kg)-induced cardiotoxicity. Fecal microbiota transplantation mirrored the effect of BMS-1 on cardiomyocyte apoptosis and cardiotoxicity, while macrophage depletion and neutralization of TNF-α and IL-1β greatly attenuated BMS-1-induced cardiotoxicity. Importantly, *Prevotella loescheii* recolonization and butyrate supplementation alleviated PD-1/PD-L1 inhibitor-related cardiotoxicity. Mechanistically, gut microbiota dysbiosis promoted M1-like polarization of colonic macrophages and the production of proinflammatory factors TNF-α and IL-1β through downregulation of PPARα-CYP4X1 axis.

**Conclusions:**

Intestinal barrier dysfunction amplifies PD-1/PD-L1 inhibitor-related cardiotoxicity by upregulating proinflammatory factors TNF-α and IL-1β in colonic macrophages via downregulation of butyrate-PPARα-CYP4X1 axis. Thus, targeting gut microbiota to polarize colonic macrophages away from the M1-like phenotype could provide a potential therapeutic strategy for PD-1/PD-L1 inhibitor-related cardiotoxicity.

**Graphical abstract:**

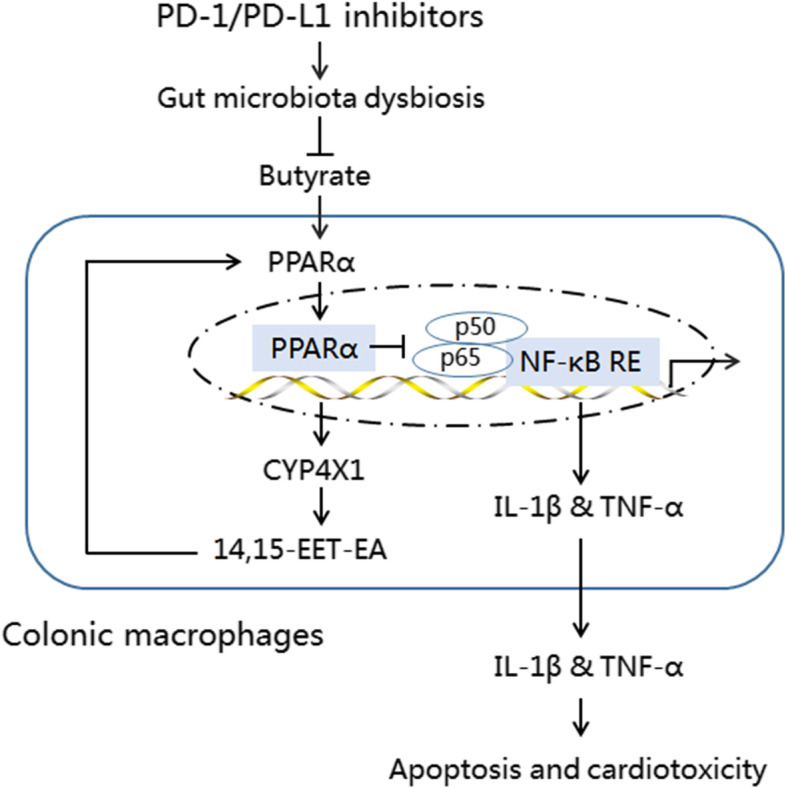

**Supplementary Information:**

The online version contains supplementary material available at 10.1186/s13046-021-02201-4.

## Background

Immune checkpoint inhibitors (ICIs) targeting programmed cell death 1 (PD-1) or its ligand 1 (PD-L1) have achieved great clinical success in antitumor therapy [1]. However, PD-1/PD-L1 inhibitors induce a wide spectrum of immune-related adverse events (irAEs), with cardiotoxicity being the most lethal adverse effect [2]. ICI-related cardiotoxicity events occur in different forms, including myocarditis, cardiomyopathy and myocardial fibrosis, and myocarditis with evidence of cardiomyocyte apoptosis is one of the most important clinical and pathological features [3, 4]. Multiple mechanisms of irAEs, including cardiotoxicity, have been proposed, especially proinflammatory cytokine release in addition to direct cytotoxic activity by T cells on nontumor cells [5, 6]. Therefore, it is essential to minimize systemic inflammation to overcome PD-1/PD-L1 inhibitor-induced cardiotoxicity, particularly myocarditis.

Alterations in gut microbial community play an important role in inflammatory response in cardiovascular disease [7, 8]. Gut microbiota metabolites short-chain fatty acids (SCFAs), such as butyrate, have anti-inflammatory properties [9]. Butyrate and its derivative phenylalanine-butyramide can protect against doxorubicin (DOX)-induced cardiotoxicity [10, 11], and a decreased abundance of microbes with the capacity to produce butyrate has been identified in the pathogenesis of DOX-related cardiotoxicity [10]. Manipulation of gut microbiota by fecal transplantation improves the efficacy of PD-1/PD-L1 inhibitors [12]. However, whether manipulation of gut microbiota and its metabolites, including butyrate, prevents PD-1/PD-L1 inhibitor-induced cardiomyocyte apoptosis and cardiotoxicity is still not clear.

Macrophages are primary producers of inflammatory mediators during autoimmune and autoinflammatory diseases [13]. A previous study showed that a PD-1 inhibitor induces cardiac injury through polarization of cardiac macrophages to an M1-like phenotype [6]. Our recent study showed that colonic macrophages, one of the most numerous leukocytes in colon, contribute to multiwalled carbon nanotube-exacerbated cardiotoxicity [14]. Conversely, flavonoid glabridin prevents DOX-induced acute cardiotoxicity by polarizing colonic macrophages away from the M1-like phenotype via gut microbiota-derived butyrate [10]. Cytochrome P450 (CYP) 4X1, an orphan CYP protein, metabolizes anandamide (N-arachidonoyl-ethanolamine, AEA) to 14,15-epoxyeicosatrienoic acids-ethanolamide (14,15-EET-EA) [15]. Our previous study showed that CYP4X1 inhibition repolarizes tumor-associated macrophages to an M1 phenotype and upregulates M1-like factors TNF-α and IL-1β [15]. However, whether CYP4X1 and colonic macrophage-derived M1-like factors TNF-α and IL-1β contribute to PD-1/PD-L1 inhibitor-related cardiotoxicity has not been explored.

Numerous immunotherapy agents, including nivolumab and pembrolizumab, have been approved by the Food and Drug Administration (FDA) for cancer treatment [16]. BMS-1, a small-molecule inhibitor of PD-1/PD-L1 interaction, possesses effects similar to anti-PD-1/PD-L1 antibodies, suggesting that it could be an alternative to antibodies used in immunotherapy [17]. In addition, compared with antibodies drugs, small-molecule PD-1/PD-L1 inhibitors offer inherent advantages in terms of their pharmacokinetics and druggability, thereby achieving better therapeutic effects and providing a promising perspective [18].

In this study, the effects of gut microbiota dysbiosis and low butyrate production on PD-1/PD-L1 inhibitor-related cardiotoxicity were first measured in mice with melanoma. We then investigated whether M1-like colonic macrophage-derived factors IL-1β and TNF-α contribute to PD-1/PD-L1 inhibitor-induced cardiomyocyte apoptosis and cardiotoxicity. Finally, we investigated whether gut microbiota dysbiosis upregulates proinflammatory factors TNF-α and IL-1β in colonic macrophages through downregulation of butyrate-CYP4X1 axis. Our results identified a novel mechanism of action of PD-1/PD-L1 inhibitor-related cardiotoxicity, and may provide a potential therapeutic target.

## Methods

### Chemicals and reagents

BMS-1 (HY-19991) and sodium butyrate (HY-B0350A) were purchased from MedChemExpress (Monmouth Junction, New Jersey, USA). Lipopolysaccharide (LPS) (L2630) was purchased from Sigma-Aldrich (St. Louis, MO, United States). Peroxisome proliferator-activated receptor α (PPARα) antagonist GW6471 (T8486) was purchased from Tocris (Bristol, UK). 14,15-EET-EA (10008599) was purchased from Cayman Chemicals (Ann Arbor, MI). Antibodies against HS-associated protein X-1 (HAX-1) (ab78939), cleaved caspase 3 (ab13847), cleaved caspase 9 (ab202068), CD68 (ab125212), iNOS (ab178945), CD206 (ab64693), CYP4X1 (ab74810) and β-actin (ab8226) were purchased from Abcam (Cambridge, MA, United States). Anti-colony-stimulating factor-1 (CSF-1) (BE0204), anti-TNF-α (BP0058) and anti-IL-1β (BE0246) antibodies were purchased from BioXCell (West Lebanon, NH, United States). Antibodies against B-cell lymphoma protein 2-associated X (Bax) (2772), B-cell lymphoma-2 (Bcl-2) (3498), p65 (8242) and phosphor-p65 (3033) were purchased from Cell Signaling Technology (Danvers, MA, USA). Antibody against PPARα (15540–1-AP) was purchased from Proteintech (Chicago, Illinois, USA). Antibodies against Claudin-1 (BM5140), Occludin (A01246–2) and proliferating cell nuclear antigen (PCNA) (BM3888) were purchased from Wuhan Boster Biological Technology (Wuhan, China). Anti-ZO-1 antibody (GB111402) was purchased from Wuhan Servicebio Biological (Wuhan, China).

### Cell cultures

Mouse melanoma B16F10 cells and the macrophage cell line RAW264.7 were obtained from the American Type Culture Collection (ATCC, Manassas, VA, USA), and cultured in Dulbecco’s modified Eagle’s medium containing 10% heat-inactivated fetal calf serum (FCS, 10100147, Gibco, Carlsbad, CA, USA) and 100 U/ml penicillin and streptomycin (10378016, Invitrogen, Carlsbad, CA, USA). Mouse cardiomyocyte HL-1 (SCC065) was purchased from Sigma–Aldrich (St. Louis, MO, USA), and cultured in Claycomb medium (51800C, Sigma–Aldrich) containing 10% heat-inactivated fetal calf serum and 100 U/ml penicillin and streptomycin. All cells were cultured at 37 °C in a humidified 5% CO_2_ atmosphere.

### Animals and treatments

C57BL/6 mice (male, 6–8 weeks old) were purchased from the Centers for Disease Control and Prevention (Hubei, China). Male *Cyp4x1* knockout (*Cyp4x1*^−/−^) mice were generated using the CRISPR/Cas9 system on a C57BL/6 background (Bioray Laboratory, Shanghai, China). The mice were acclimatized for 1 week to adapt to the new environment before the experiment. The animals were housed in polypropylene cages with food and water available ad libitum. The room temperature was maintained at 18–22 °C under a 12 h light/12 h dark cycle. The mice were caged individually to avoid any effects of cohousing on microbiota composition.

For PD-1/PD-L1 inhibitor BMS-1 treatment experiment, B16F10-luciferase cells (5 × 10^5^) were injected into the right flank of the C57BL/6 mice. When the tumors reached a size of approximately 100 mm^3^, the mice were randomly divided into three groups (*n* = 10): BMS-1 (5 and 10 mg/kg) and control groups. The mice were intraperitoneally injected with BMS-1 (0, 5 and 10 mg/kg) every 2 days for a total of 6 times. Body weight and food intake were measured per animal. Tumor growth was detected by a luciferase-based noninvasive bioluminescence imaging system (In-Vivo Xtreme II, Bruker). The melanoma images were analyzed by quantification of total photon flux of each tumor using Molecular Imaging Software. The mice were euthanized 24 h after the last injection of BMS-1, and then peripheral blood, tumors, colonic and cardiac tissues were collected and analyzed. The mice in the control group (*n* = 10) were intraperitoneally injected with an equivalent volume of phosphate buffer saline (PBS). The doses (5 and 10 mg/kg) of BMS-1 used in this study were based on a published study [19] and our preliminary experiments.

Fecal microbiota transplantation (FMT) was performed as described [14]. Briefly, the mice were pretreated with a cocktail of broad-spectrum antibiotics (Abx), including 0.5 g/L vancomycin, 0.5 g/L neomycin sulfate and 0.5 g/L primaxin in the drinking water for 2 weeks to clear the gut microbiota. Next, the mice were orally administered a fecal suspension (300 μL per mouse) derived from BMS-1 (5 and 10 mg/kg) or PBS-treated mice every 2 days for a total of 6 times.

The bacterial colonization or exogenous metabolite supplementation experiments were performed as previously described [10]. Briefly, a cocktail of lyophilized *Prevotella loescheii* (*P. loescheii*) (ATCC 15930) was resuspended in phosphate-buffered saline at 5 × 10^9^ colony forming units (CFUs)/ml. The mice (*n* = 10) were orally administered with sodium butyrate (1 g/kg/day) or cocktail (200 μl/mouse, every 2 days) for 1 week. The dose of sodium butyrate used in the present study was based on published study [10] and our preliminary experiments.

Macrophage depletion experiments were performed with an anti-CSF-1 antibody as previously described [20]. Briefly, the mice (*n* = 10) were intraperitoneally administered with 50 mg/kg anti-CSF-1 antibody 24 h before BMS-1 injection, followed by repeated injections of 25 mg/kg anti-CSF-1 antibody every 5 days.

IL-1β and TNF-α neutralization experiments were performed as previously described [14]. Briefly, the mice (*n* = 10) were administered intraperitoneally with anti-TNF-α antibody (100 μg), anti-IL-1β antibody (100 μg) or their combination every 2 days for a total of 3 times during the PD-1/PD-L1 inhibitor treatment. The control mice received 100 μg IgG as an isotype control [21], and no IgG-related side effects were observed during the experiment.

### Fecal microbiota analysis by 16S rRNA sequencing

Fecal microbiota analysis by 16S rRNA sequencing was based on a previous description [14]. Briefly, fresh stool pellets from the PD-1/PD-L1 inhibitor BMS-1 (5 and 10 mg/kg) and PBS-treated mice were obtained before the mice were euthanized, and immediately frozen in liquid nitrogen. Genomic DNA from fecal samples (50–100 mg) was extracted using the TIANamp stool DNA kit (TIANGEN Biotech Co., Ltd., Beijing, China) according to the manufacturer’s instructions. The concentration and purity of the extracted bacterial DNA were measured using a NanoDrop 2000C spectrophotometer (Thermo Fisher Scientific, Waltham, USA). The 16S rRNA gene in the fecal DNA samples was amplified by polymerase chain reaction (PCR) targeting the hypervariable V3-V4 region of the 16S rRNA gene of bacteria with primers 338-F (5′-GTGCCAGCMGCCGCGGTAA-3′) and 806-R (5′-GGACTACHVGGGTWTCTAAT-3′). Sequencing was performed by an Illumina MiSeq PE300 system (OE Biotech Co., Ltd.). As an added quality control measure, the software ckage MacQIIME (version 1.9.1) pipeline was used to filter out and discard poor-quality reads using the default settings [20]. The sequences were further clustered into operational taxonomic units or phylotypes (OTUs) at 97% identity using QIIME and cdhit. OTUs were assigned to the closest taxonomic neighbors and relative bacterial species using Seqmatch and Blastall. Principle coordinate analysis (PCoA) projections were visualized using Emperor 0.9.4.

### Analysis of short-chain fatty acids in the feces

SCFAs in the feces from BMS-1 (5 and 10 mg/kg) and control groups were analyzed by high-performance liquid chromatography (HPLC) based on a previous description [10]. Then, 400 μL of HCl was added to the fecal homogenates to preserve the volatile SCFAs and they were centrifuged at 14,000 g to evenly suspend the fecal mass. The resulting supernatants were then passed through a 0.22 μm syringe filter to remove bacterial cells and any debris. SCFA analyses were performed using an Agilent Technologies 1290 Infinity II and a 300 SB C18 column (1.8 μm, 2.1 × 100 mm) with a guard column, and 0.01 M H_2_SO_4_ was used as the mobile phase. SCFAs were identified by comparing sample peak retention times to a standard mixture of acetate, propionate, butyrate, valerate, and hexanoate.

### Isolation of peritoneal and colonic macrophages

Peritoneal macrophages (PNMSs) were isolated and grown as described previously [14]. Briefly, the mice treated with the PD-1/PD-L1 inhibitor BMS-1 (10 mg/kg) were sacrificed, and 5 mL of heat-inactivated PBS was injected into the abdomen. Then, the abdomen was massaged gently for 3 min. The peritoneal fluid was drawn back. After centrifugation at 1500 g for 10 min, the cell pellets were suspended in DMEM (supplemented with 10% (v/v) bovine calf serum, 100 U/mL penicillin and 100 U/mL streptomycin) and then allowed to adhere for 3 h at 37 °C in a humidified incubator containing 5% CO_2_. After 4 h of incubation, nonadherent cells were removed by washing twice with PBS, and then freshly prepared medium was added.

Colonic macrophages were isolated by flow cytometry as previously described [20]. Briefly, colonic tissues from BMS-1 (5 and 10 mg/kg), *Cyp4x1*^*−/−*^, *P. loescheii*, butyrate and control groups were cut into 0.5 cm pieces, and then incubated with 0.25% trypsin at 37 °C for 30 min. The cell suspension was passed through a 100 μm strainer and stained with PerCPCy5.5-conjugated anti-CD11c antibody (ab111469, Abcam, Cambridge, MA, United States), FITC-conjugated anti-F4/80 antibody (ab105155, Abcam, Cambridge, MA, United States) and PE-conjugated anti-CD11b antibody (ab269361, Abcam, Cambridge, MA, United States). The stained cells were then sorted using a FACS Aria Cell Sorter (BD Biosciences). The purity of the colonic macrophage was > 95%. Freshly isolated colonic macrophages were incubated in 12-well plates at 80 to 90% confluence, and resupplied with serum-free RPMI 1640 media for 24 h. The supernatant and colonic macrophages were collected for further experiments.

### Transfection with PPARα siRNA

The PPARα siRNA transfection experiment was performed as previously described [22]. siRNA targeting PPARα (sc-36,308) was obtained from Santa Cruz Biotech, Inc. (Dallas, TX, USA), and transfection was performed using Santa Cruz’s siRNA transfection reagent (sc-29,528), transfection medium (sc-36,868), and dilution buffer (sc-29,527) according to manufacturer’s instructions. Briefly, PNMS and RAW264.7 cells were seeded into six-well plates in 2 mL medium. For each well, 0.8 mL transfection mixture containing 200 nmol of PPARα siRNA was added and incubated at 37 °C for 7 h. After incubation, the cells were maintained with 1 mL of normal growth medium without removing siRNA mixture at 37 °C for 24 h. Then, the medium was replaced with fresh normal growth medium and the cells were incubated for an additional 24 h. After preparation of the cell lysates, depletion of PPARα expression was confirmed by qPCR and western blot analysis.

### Preparation of conditioned media (CM)

The CM was prepared as previously described [15]. In brief, PNMS and RAW264.7 cells were stimulated with LPS (1 μM), butyrate (5 mM), GW6471 (25 μM), 14,15-EET-EA (20 mg/ml), PPARα siRNA or vehicle (DMSO or control siRNA) for 12 h. The culture supernatants were harvested, and then subjected to centrifugation through an Amicon Ultra4 filter to remove any traces of butyrate, GW6471, 14,15-EET-EA or PPARα siRNA. The retentate was collected as CM.

### Laser Doppler analysis of mesenteric perfusion

The mesenteric perfusion was measured by laser Doppler analysis as previously described [23]. Briefly, the mice treated with the PD-1/PD-L1 inhibitor BMS-1 (0, 5 and 10 mg/kg) as described above were anesthetized with 3% or 0.6% pentobarbital and placed on a heating pad (37 °C). An incision in the abdomen was made, and the distal ileum and its accompanying mesentery were exposed for in vivo observation of the microcirculation. The mesenteric vessels were carefully separated, and Krebs-Henseleit solution (37 °C) saturated with a mixture of gases (95% N_2_ and 5% CO_2_) was used to maintain the mesentery warm and moist. Mesenteric perfusion of each animal was blindly measured using a laser Doppler analyzer with a CCD camera (SXG40c, Baumer, Germany). The mesenteric perfusion in arbitrary perfusion units was monitored graphically. The mice were euthanized after completion of the treatment.

### Statistics

Data are presented as mean ± standard error of the mean (SEM). Normal distribution of the data was assessed by the Kolmogorov–Smirnov test. For normally distributed data, Student’s t–test was used for two-group comparisons, and one-way ANOVA followed by Student–Newman–Keul’s test was used for multiple-group comparisons [10]. The statistical significance of changes over time was evaluated by one-way repeated-measures ANOVA followed by Bonferroni’s post hoc test. The data of microbiome abundance were analyzed by the nonparametric Mann–Whitney *U* test corrected for multiple comparisons. The predominance of bacterial communities between groups was analyzed using the linear discriminant analysis (LDA) effect size (LEfSe) (LDA score (log10) = 2.0 as the cutoff value) [24]. Statistical analysis was carried out by GraphPad Prism 8.0.2 software [20], and statistical significance was defined as *P* < 0.05.

## Results

### BMS-1 induces cardiomyocyte apoptosis and cardiotoxicity in mice with melanoma

**Fig. 1:**
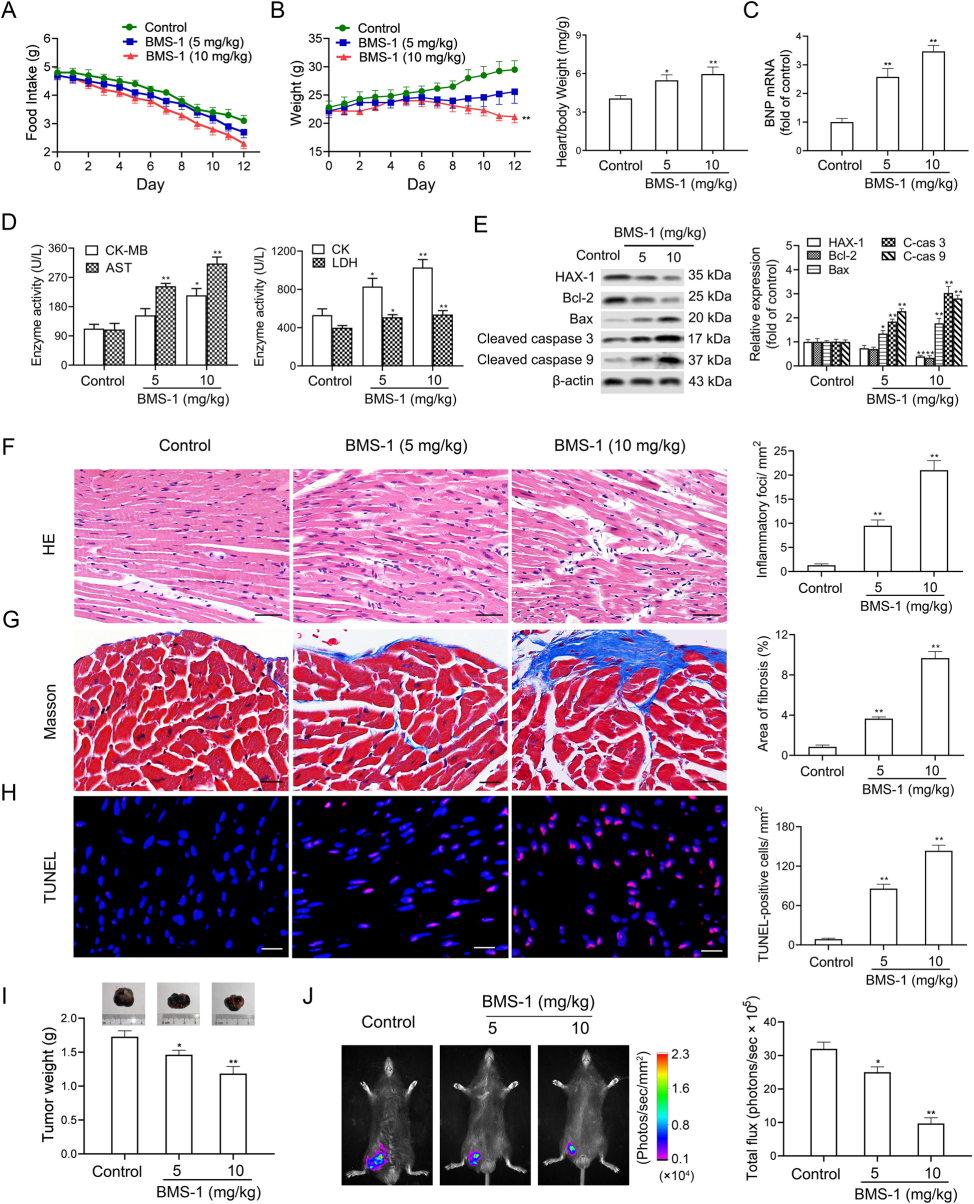
BMS-1 induces cardiomyocyte apoptosis and cardiotoxicity in mice with melanoma. In the B16F10 melanoma model, the mice were intraperitoneally administered with the PD-1/PD-L1 inhibitor BMS-1 (0, 5 and 10 mg/kg) every 2 days for 6 times (*n* = 10). **A** Changes in food intakes. **B** Changes in body weights and the ratio of heart/body weight. Statistical significance of changes over time was evaluated by one-way repeated measures ANOVA followed by Bonferroni’s post hoc test. **C** The expression of brain natriuretic peptide (BNP) in cardiac tissues was measured by qPCR. **D** The serum levels of creatine kinase-MB (CK-MB), aspartate transaminase (AST), creatine kinase (CK) and lactate dehydrogenase (LDH). **E** The protein expression of hematopoietic-substrate-1 associated protein X-1 (HAX-1), B lymphocytoma-2 gene (Bcl-2), Bcl-2 associated X protein (Bax), cleaved caspase-3 and cleaved caspase-8 in cardiac tissues. **F**-**G** Representative images of hematoxylin and eosin (HE) staining and Masson staining of cardiac tissues and corresponding quantification analysis. Scale bars, 20 μm. **H** Representative images of TUNEL assay of cardiac tissues and corresponding quantification analysis. Scale bars, 50 μm. **I** Tumor weight. **J** Representative bioluminescence images of mice bearing tumors on day 15 after implantation. Signal intensity was measured as photon flux (photons/second) and coded to a color scale. The values are presented as the mean ± standard error of the mean. ^*^P < 0.05, ^**^P < 0.01 vs. control

The food intake and body weights of all of the mice were monitored throughout the study. The food intake by the mice treated with the PD-1/PD-L1 inhibitor BMS-1 (5 and 10 mg/kg) showed a decreasing trend compared with the control, although there was no significant difference among the three groups (Fig. 1A). BMS-1 (5 and 10 mg/kg) decreased the weight but increased the ratio of heart/body weight of the mice (Fig. 1B). Cardiotoxicity is defined as heart injury (functional or structural) related to cancer treatment [25]. A previous study showed that brain natriuretic peptide (BNP), an indicator of cardiac malfunction, is significantly elevated in ICI-associated cardiotoxicity [26]. As expected, BMS-1 (10 mg/kg) significantly increased the BNP level (Fig. 1C). Similar results were obtained in the activities of myocardial enzymes including CK-MB, AST, CK and LDH in the peripheral blood of the mice treated with BMS-1 (Fig. 1D). In addition, BMS-1 (10 mg/kg) significantly decreased the levels of antiapoptotic proteins including HAX-1 and Bcl-2, but it increased the levels of pro-apoptotic proteins including Bax, cleaved caspase 3 and cleaved caspase 9 (Fig. 1E). BMS-1 (5 and 10 mg/kg) also increased inflammatory infiltration, interstitial fibrosis and the number of terminal deoxynucleotidyl transferase dUTP nick end labeling (TUNEL)-positive cardiomyocytes in the cardiac tissues compared with the control (Fig. 1F-H). Similar to previous studies [17], BMS-1 (5 and 10 mg/kg) inhibited melanoma growth, as demonstrated by a decreased tumor weight and luciferase intensity (Fig. 1I and J). These data suggested that the PD-1/PD-L1 inhibitor BMS-1 induces cardiomyocyte apoptosis and cardiotoxicity in mice with melanoma.

### BMS-1 induces intestinal mucosal barrier injury in mice with melanoma

**Fig. 2:**
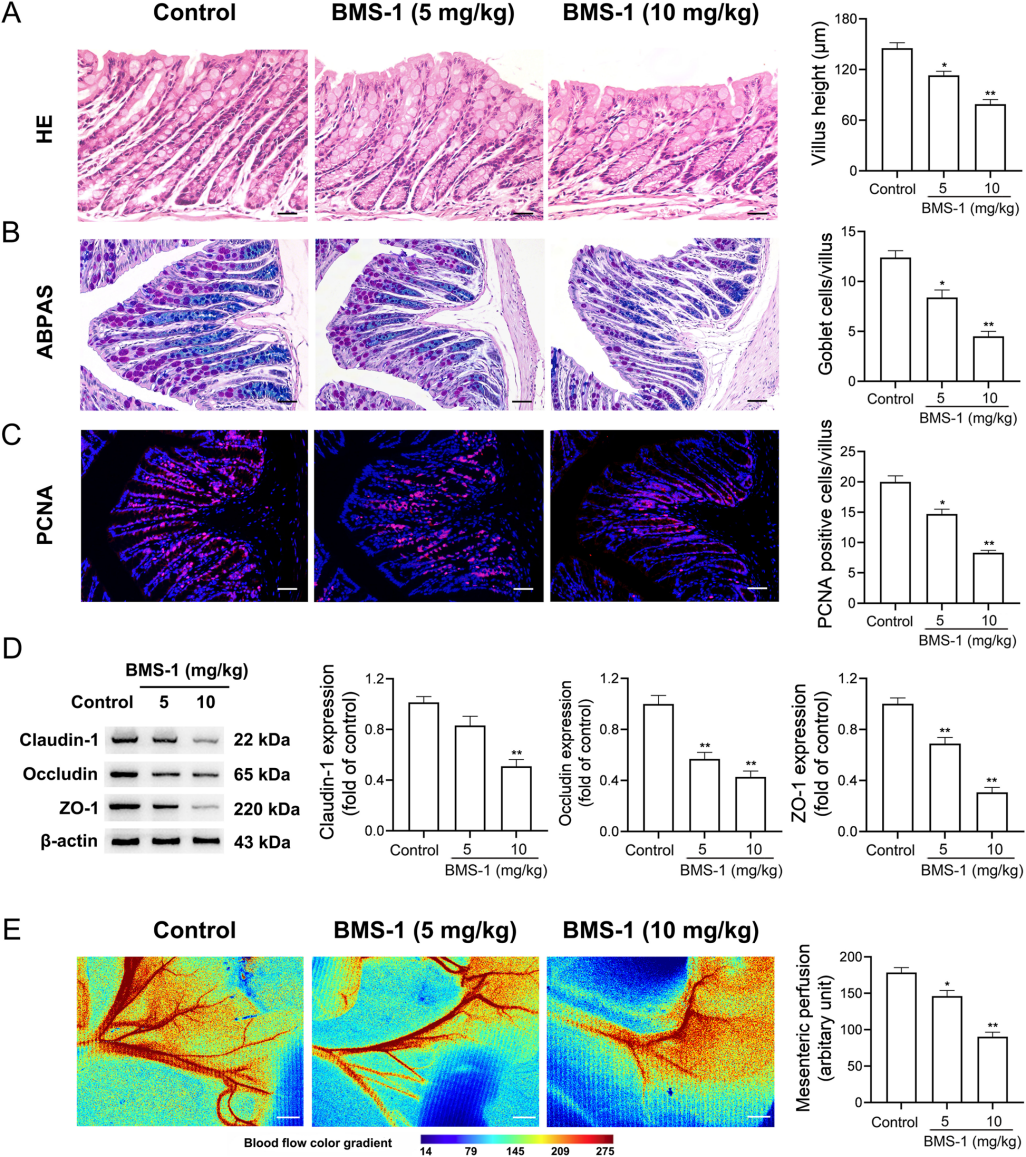
BMS-1 induces intestinal barrier injury in mice with melanoma. In the B16F10 melanoma model, the mice were intraperitoneally administered with the PD-1/PD-L1 inhibitor BMS-1 (0, 5 and 10 mg/kg) every 2 days for 6 times (*n* = 10). **A** Representative images of hematoxylin and eosin (HE) staining of colonic tissues and corresponding quantification analysis. Scale bars, 100 μm. **B** Representative images of Alcian blue-periodic acid-Schiff (ABPAS) of colonic tissues and corresponding quantification analysis. Scale bars, 100 μm. **C** Representative images of proliferating cell nuclear antigen (PCNA) and corresponding quantification analysis. Scale bars, 100 μm. **D** Representative western blot images of Claudin-1, Occludin and ZO-1 in colonic tissues and corresponding quantification analysis. **E** Representative images of laser Doppler analysis and corresponding quantification analysis. Scale bar, 2 mm. The values are presented as the mean ± standard error of the mean. ^*^P < 0.05, ^**^P < 0.01 vs. control

Intestinal barrier dysfunction including mechanical barrier (intestinal mucosal epithelia) and biological barrier (gut microbiota) injury contributes to impaired cardiac function [27]. Therefore, the colonic mucosal integrity was evaluated. As shown in Fig. 2A-D, BMS-1 (5 and 10 mg/kg) decreased the villus height and the number of goblet cells along with the number of proliferating cell nuclear antigen (PCNA)-positive cells, and it downregulated the expression of tight junction proteins Claudin-1, Occludin and ZO-1 in the colonic tissues compared with the control. Next, we determined the mesenteric perfusion scores, and found that BMS-1 (5 and 10 mg/kg) decreased the mesenteric perfusion (Fig. 2E). These data suggested that BMS-1 induces intestinal mucosal barrier injury in mice with melanoma.

### Gut microbiota dysbiosis contributes to PD-1/PD-L1 inhibitor-related cardiotoxicity

**Fig. 3:**
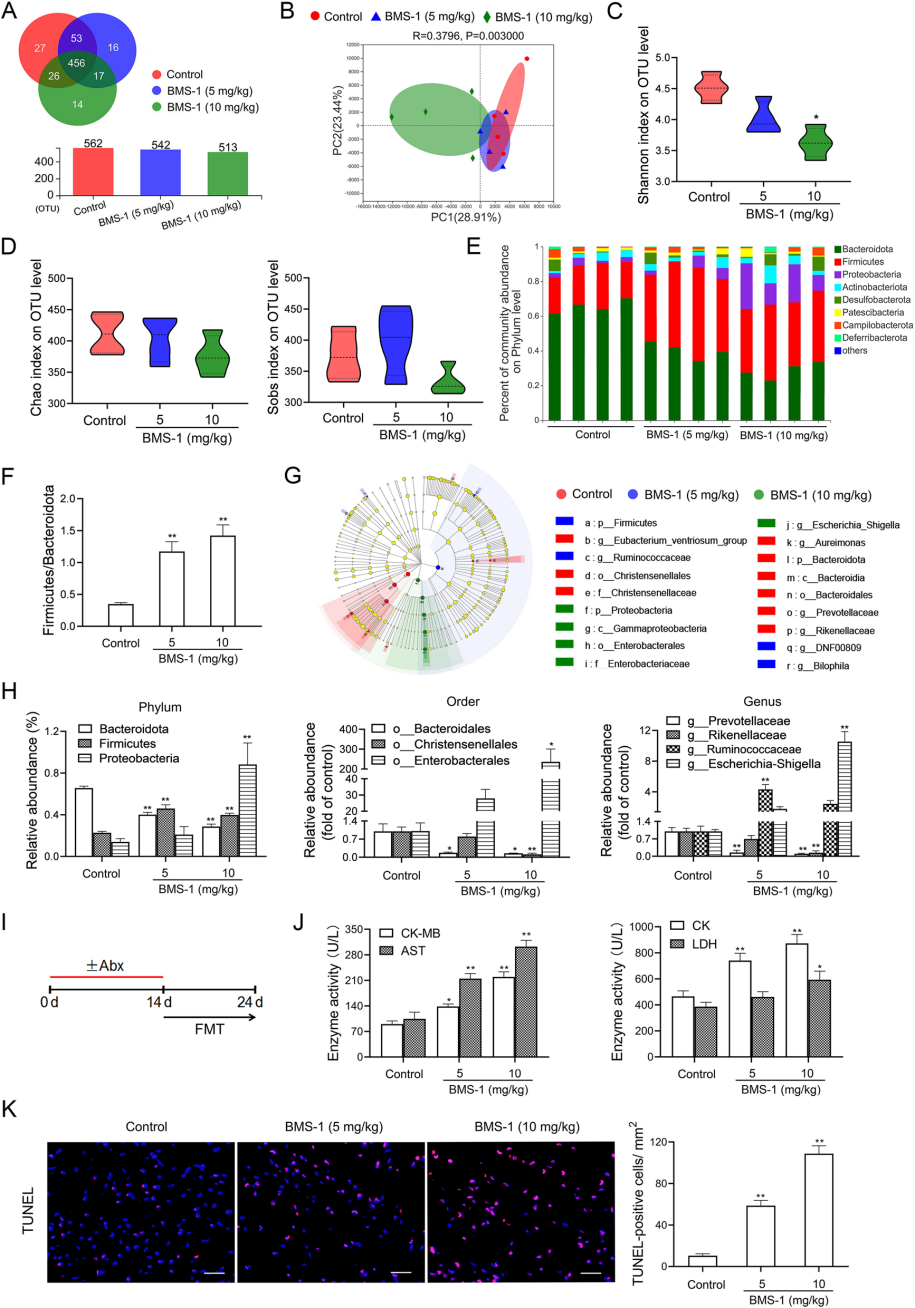
Gut microbiota dysbiosis contributes to PD-1/PD-L1 inhibitor-related cardiotoxicity. In the B16F10 melanoma model, the mice were intraperitoneally administered with the PD-1/PD-L1 inhibitor BMS-1 (0, 5 and 10 mg/kg) every 2 days for 6 times (*n* = 4). **A** The total fecal bacterial load and category. **B** Principal coordinates analysis (PCoA) plot of bacterial beta diversity. **C**-**D** Alpha diversity indices (Shannon index, Chao index and Sobs index) of OTU distribution in each sample. **E** The relative taxonomic abundance at the phylum level of gut microbiota. **F** The ratios of Firmicutes/Bacteroidota. **G** Taxonomic cladogram from linear discriminant analysis effect size (LEfSe). Dot size is proportional to the abundance of the taxon. **H** The relative abundances of differentially expressed bacteria at phylum, order and genus level. **I** In the B16F10 melanoma model, the mice pretreated with a cocktail of broad-spectrum antibiotic (Abx) were orally administrated with a supernatant of fresh stool pellets derived from BMS-1 (5 and 10 mg/kg) or vehicle-treated mice every 2 days for 6 times (*n* = 10). **J** The serum levels of creatine kinase-MB (CK-MB), aspartate transaminase (AST), creatine kinase (CK) and lactate dehydrogenase (LDH). **K** Representative images of TUNEL assay of cardiac tissues and corresponding quantification analysis. Scale bars, 50 μm. The values are presented as the mean ± standard error of the mean. ^*^P < 0.05, ^**^P < 0.01 vs. control. OTUs, Operational Taxonomic Units

Next, we sequenced the bacterial 16S rRNA in the feces to assess the intestinal biological barrier. The rarefaction curves of the bacterial community reached a saturation plateau, indicating that the sequencing depth was sufficient to represent the majority of microbe species (Fig. S1). Although BMS-1 (5 and 10 mg/kg) did not significantly influence the total fecal bacterial load or category (Fig. 3A), microbial beta diversity analysis based on principal coordinate analysis showed an obvious dissimilarity in gut microbiota constitution, especially between the BMS-1 (10 mg/kg) and control groups (Fig. 3B). This was further supported by microbial alpha diversity analysis, as calculated by decreased the Shannon index, without influencing the Chao index and Sobs index (Fig. 3C and D). Figure 3E showed that BMS-1 significantly altered the abundance of bacterial phyla. The ratio between Firmicutes and Bacteroidetes, a marker of gut dysbiosis [10], was significantly increased by BMS-1 (5 and 10 mg/kg) (Fig. 3F). LEfSe was used to define the differentially expressed bacteria (Fig. 3G). At the phylum level, BMS-1 (5 and 10 mg/kg) significantly depleted Bacteroidota. Conversely, the abundance of Firmicutes and Proteobacteria was significantly increased in BMS-1 (10 mg/kg)-treated mice compared with the control. At the order level, BMS-1 dose (5 and 10 mg/kg)-dependently depleted Bacteroidales and Christensenellales but enriched Enterobacterales. At the genus level, a depletion of *Prevotellaceae* and *Rikenellaceae* genus and an enrichment of *Escherichia-Shigella* were observed in BMS-1 (10 mg/kg)-treated mice compared with the control. The abundance of *Ruminococcaceae* genus was significantly increased in BMS-1 (5 mg/kg)-treated mice as compared with the control, although there was no significant difference between the BMS-1 (10 mg/kg) and control groups (Fig. 3H). To test the role of the gut microbiota in BMS-1-induced cardiotoxicity, we performed an FMT experiment in which gut microbiota-depleted mice were reconstituted with the gut microbiota of BMS-1 (5, 10 mg/kg)-treated mice (Fig. 3I). Unexpectedly, FMT from BMS-1-treated mice significantly induced the leakage of myocardial enzymes (CK-MB, AST, CK and LDH) and the apoptosis of cardiomyocytes (Fig. 3J and K). These data suggested that gut microbiota dysbiosis contributes to PD-1/PD-L1 inhibitor-related cardiotoxicity.

### *P. loescheii* colonization and butyrate supplementation alleviate PD-1/PD-L1 inhibitor-related cardiotoxicity

**Fig. 4:**
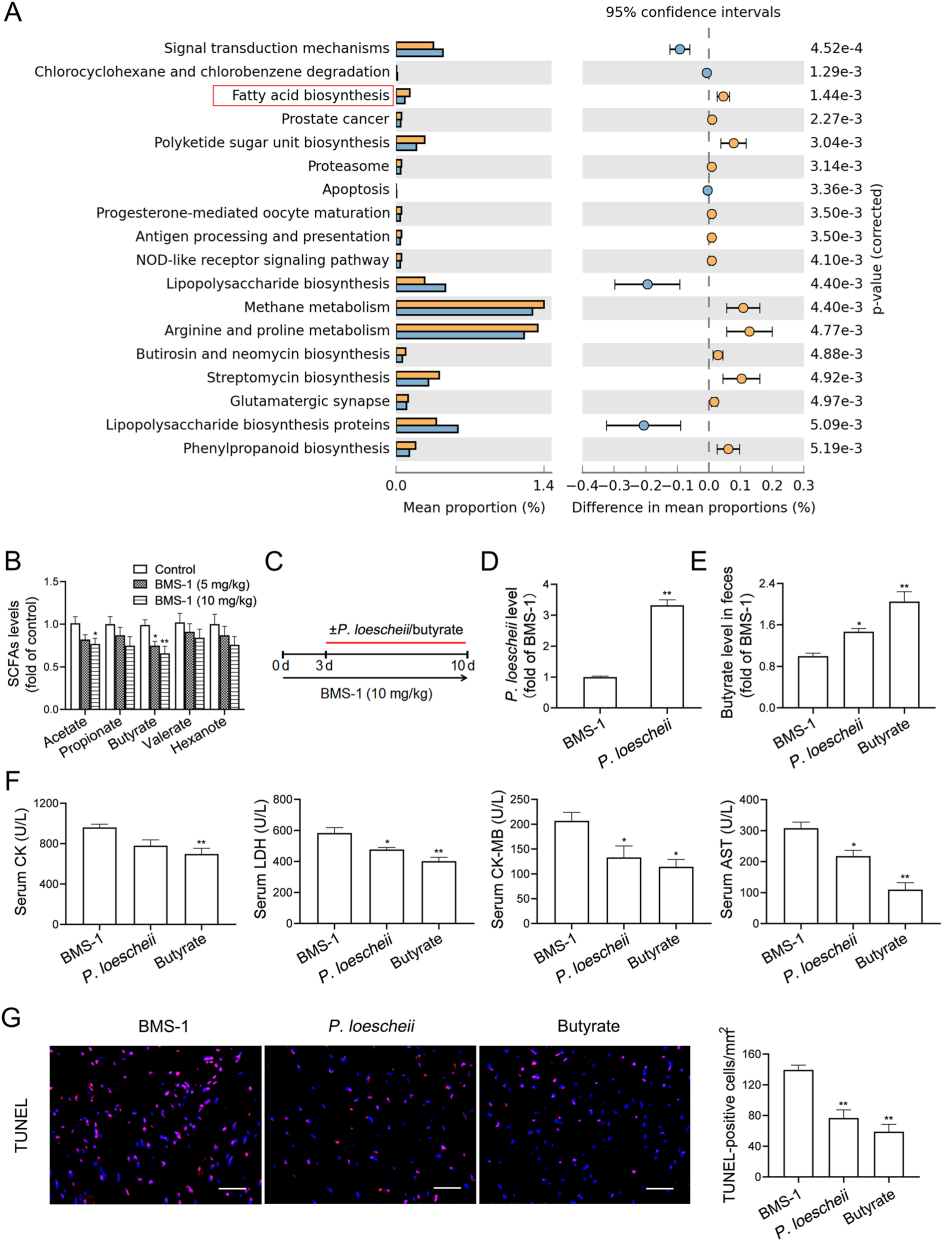
*P. loescheii* colonization and butyrate supplementation alleviate PD-1/PD-L1 inhibitor-related cardiotoxicity. In the B16F10 melanoma model, the mice were intraperitoneally administered with the PD-1/PD-L1 inhibitor BMS-1 (0, 5 and 10 mg/kg) every 2 days for 6 times (*n* = 4). **A** Significant Kyoto Encyclopedia of Genes and Genomes (KEGG) pathways at level 3 for the fecal microbiome of BMS-1 (10 mg/kg) and the control group were identified by STAMP software. **B** The levels of SCFAs in the feces were determined by HPLC. **C** In the B16F10 melanoma model, the C57BL/6 mice were orally administrated with *Prevotellaceae loescheii* (*P. loescheii*) (1 × 10^8^ CFU/mouse/every 2 days) or sodium butyrate (1 g/kg) for 1 week during BMS-1 (10 mg/kg) treatment (*n* = 10). **D** The relative level of *P. loescheii*. **E** The level of butyrate in the feces was determined by HPLC. **F** The serum levels of creatine kinase-MB (CK-MB), aspartate transaminase (AST), creatine kinase (CK) and lactate dehydrogenase (LDH). **G** Representative images of TUNEL assay of cardiac tissues and corresponding quantification analysis. Scale bars, 50 μm. The values are presented as the mean ± standard error of the mean. ^*^P < 0.05, ^**^P < 0.01 vs. control or BMS-1

KEGG pathway analysis was performed to detect the relative abundances of functional genes in gut microbiota. Remarkably, the fatty acid synthesis pathway was predominantly downregulated in BMS-1 (10 mg/kg)-treated mice compared with the control (Fig. 4A). Gut microbiota metabolites SCFAs mediate anti-inflammatory effects in numerous inflammatory diseases, and low microbial SCFA production is linked to heart failure [10]. Thus, we measured SCFAs in the feces from BMS-1 and control mice, and found that BMS-1 (5 and 10 mg/kg) significantly decreased butyrate level, while acetate, propionate, valerate and hexanote remained unchanged (Fig. 4B). Next, we supplied *P. loescheii*, one of the main SCFA-producing strain in *Prevotellaceae* [28], or butyrate to BMS-1 (10 mg/kg)-treated mice (Fig. 4C), and the efficacy of *P. loescheii* colonization was confirmed by increased levels of *P. loescheii* (Fig. 4D) and butyrate in the feces (Fig. 4E). Obviously, *P. loescheii* colonization and butyrate supplementation attenuated BMS-1-induced myocardial enzyme leakage and myocardial apoptosis (Fig. 4F and G). These data suggested that *P. loescheii* colonization and butyrate supplementation alleviate PD-1/PD-L1 inhibitor-related cardiotoxicity.

### M1-like colonic macrophages and their factors IL-1β and TNF-α contribute to PD-1/PD-L1 inhibitor-related cardiotoxicity

**Fig. 5:**
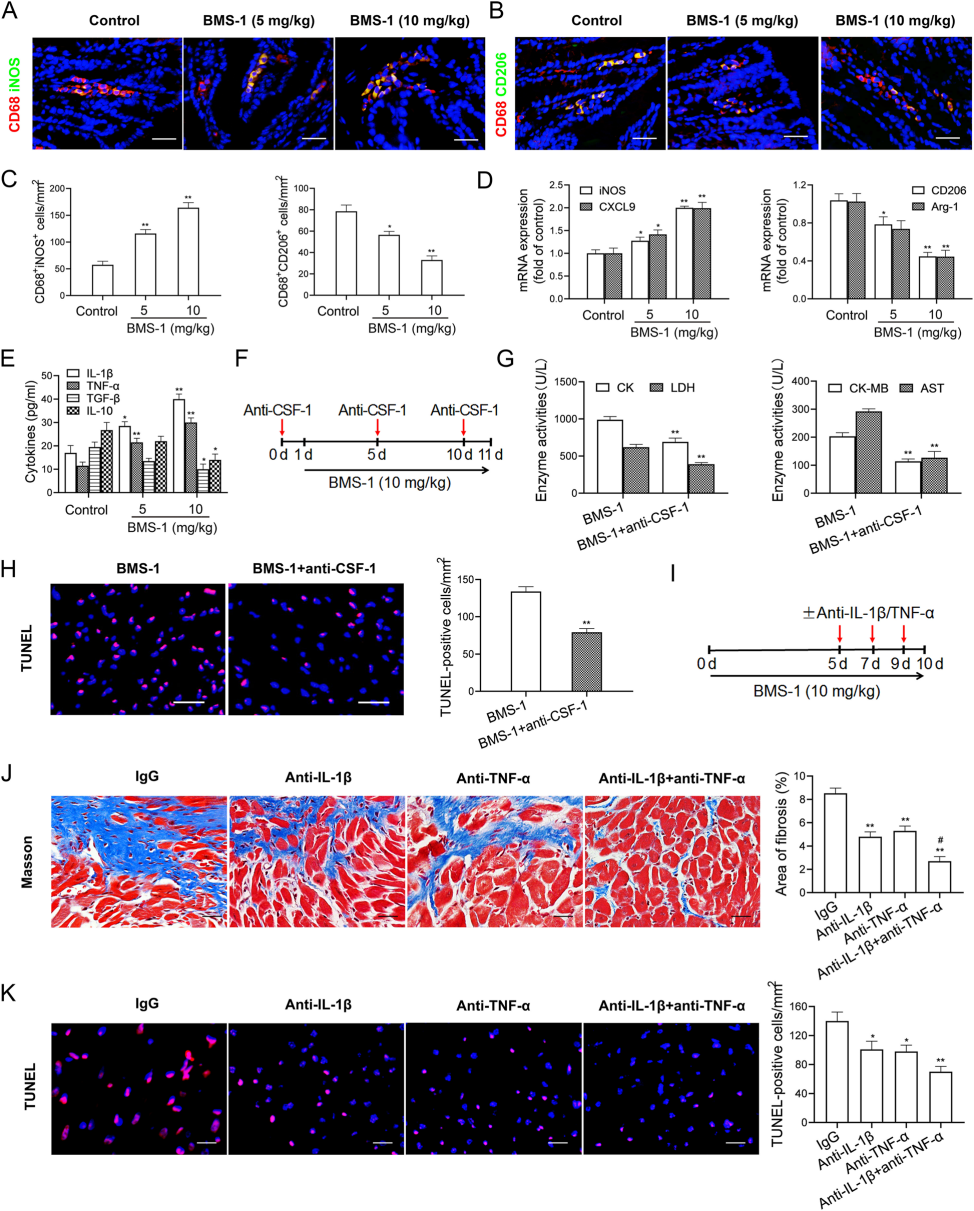
M1-like colonic macrophages and their factors IL-1β and TNF-α contribute to PD-1/PD-L1 inhibitor-related cardiotoxicity. In the B16F10 melanoma model, the mice were intraperitoneally administered with the PD-1/PD-L1 inhibitor BMS-1 (0, 5 and 10 mg/kg) every 2 days for 6 times (*n* = 10). **A**-**C** Representative images of immunostaining analysis of CD68^+^iNOS^+^ and CD68^+^CD206^+^ cells in colonic tissues and corresponding quantitative analysis. Scale bars, 20 μm. **D** M1 marker (iNOS and CXCL9) and M2 marker (CD206 and Arg-1) in colonic macrophages were measured by qPCR. **E** The production of M1 factors (IL-1β and TNF-α) and M2 factors (TGF-β and IL-10) in colonic macrophages were determined by ELISA. **F** In the B16F10 melanoma model, the mice were administrated intraperitoneally with 50 mg/kg anti-CSF-1 antibody 24 h before BMS-1 (10 mg/kg) injection, and followed by repeated injections of 25 mg/kg every 5 d to deplete colonic macrophages (*n* = 10). **G** The serum levels of creatine kinase-MB (CK-MB), aspartate transaminase (AST), creatine kinase (CK) and lactate dehydrogenase (LDH). **H** Representative images of TUNEL assay of cardiac tissues and corresponding quantification analysis. Scale bars, 50 μm. **I** In the B16F10 melanoma model, the mice were administrated intraperitoneally with anti-IL-1β antibody (100 μg), anti-TNF-α antibody (100 μg) or their combination (IgG as control) every 2 days for 3 times during BMS-1 (10 mg/kg) injection (*n* = 10). **J** Representative images of Masson staining of cardiac tissues and corresponding quantification analysis. Scale bars, 20 μm. **K** Representative images of TUNEL assay of cardiac tissues and corresponding quantification analysis. Scale bars, 50 μm. The values are presented as the mean ± standard error of the mean. ^*^P < 0.05, ^**^P < 0.01 vs. control or BMS-1 or IgG, ^#^*P* < 0.05, ^##^*P* < 0.01 vs. anti-TNF-α

A previous study showed that a PD-1 inhibitor induces M1-like polarization of cardiac macrophages [6]. We found that BMS-1 (5 and 10 mg/kg) significantly increased the number of M1-like (CD68^+^iNOS^+^) cells and upregulated M1 genes (iNOS and CXCL9), but decreased the number of M2-like (CD68^+^CD206^+^) cells and downregulated M2 genes (CD206 and Arg-1) (Fig. 5A-D). In addition, BMS-1 significantly increased the levels of IL-1β and TNF-α but not TGF-β and IL-10 in colonic macrophages (Fig. 5E). To determine whether BMS-1 induced cardiotoxicity through the alteration of the colonic macrophage phenotype, we depleted colonic macrophages with an anti-CSF-1 antibody (IgG as a control) (Fig. 5F). As shown in Fig. 5G and H, anti-CSF-1 antibody significantly decreased the levels of myocardial enzymes (CK, LDH, CK-MB and AST) in peripheral blood and the number of TUNEL-positive cells in the cardiac tissues from the mice treated with BMS-1 (10 mg/kg). Next, we used antibodies against IL-1β and TNF-α to block their effects (Fig. 5I) and found that the anti-IL-1β or anti-TNF-α antibody partly abolished BMS-1-induced cardiotoxicity, as shown by decreased interstitial fibrosis and myocardial apoptosis in cardiac tissues. Importantly, a combined block of IL-1β and TNF-α showed better efficacy than a single block in attenuating PD-1/PD-L1 inhibitor-related cardiotoxicity (Fig. 5J and K). These data suggested that the M1-like colonic macrophage-derived factors IL-1β and TNF-α contribute to PD-1/PD-L1 inhibitor-induced cardiomyocyte apoptosis and cardiotoxicity.

### *P. loescheii* colonization and butyrate supplementation downregulate IL-1β and TNF-α in colonic macrophages

**Fig. 6:**
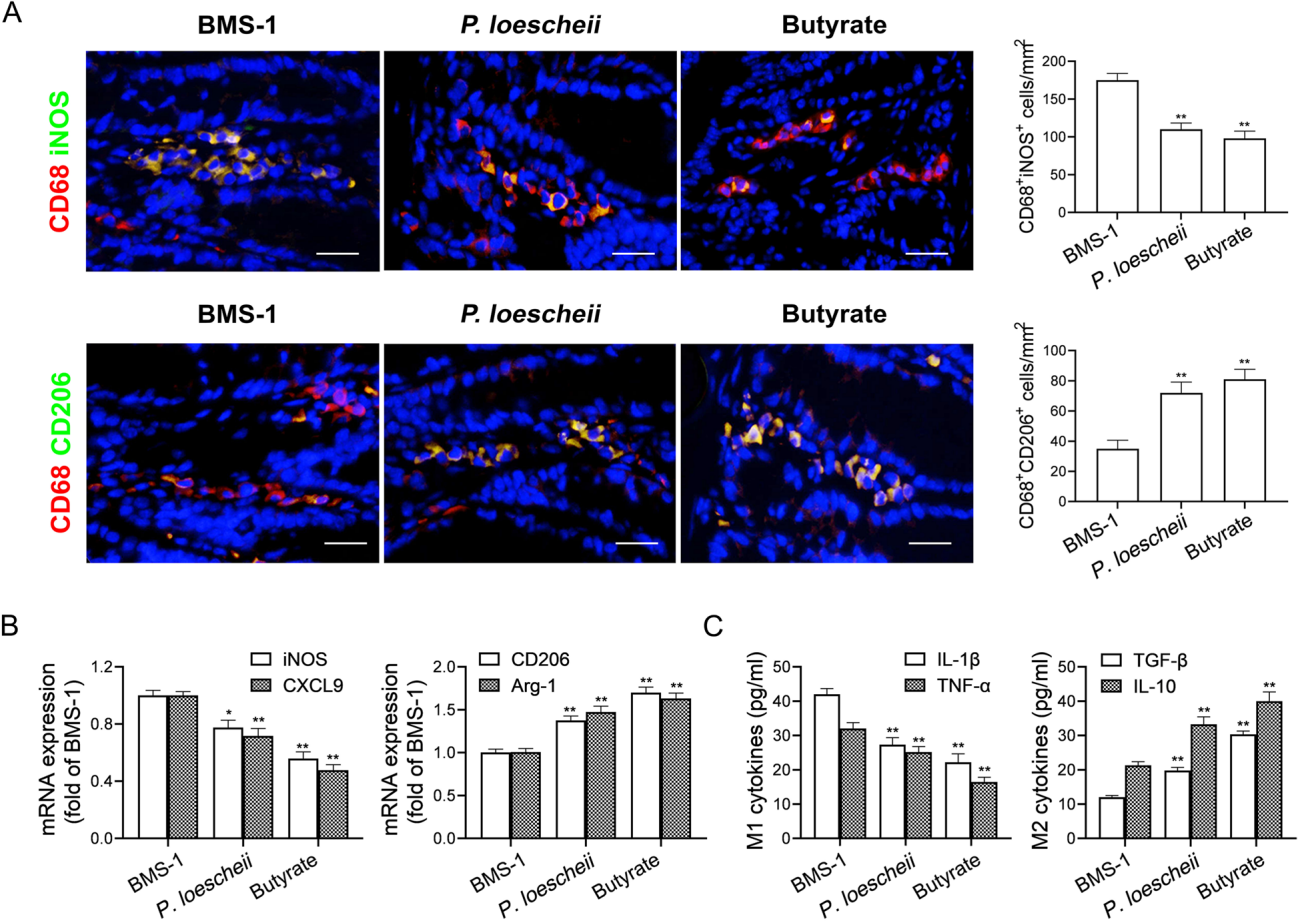
*P. loescheii* colonization and butyrate supplementation downregulate IL-1β and TNF-α in colonic macrophages. In the B16F10 melanoma model, the C57BL/6 mice were orally administrated with *Prevotellaceae loescheii* (*P. loescheii*) (1 × 10^8^ CFU/mouse/every 2 days) or sodium butyrate (1 g/kg) for 1 week during BMS-1 (10 mg/kg)-treatment (*n* = 10). **A** Representative images of immunostaining analysis of CD68^+^iNOS^+^ and CD68^+^CD206^+^ cells in colonic tissues and corresponding quantitative analysis. Scale bars, 20 μm. **B** M1 marker (iNOS and CXCL9) and M2 marker (CD206 and Arg-1) in colonic macrophages were measured by qPCR. **C** The M1 factors (IL-1β and TNF-α) and M2 factors (TGF-β and IL-10) in colonic macrophages were determined by ELISA. The values are presented as the mean ± standard error of the mean. ^*^P < 0.05, ^**^P < 0.01 vs. BMS-1

Our previous study showed that decreased butyrate level in feces reprogrammed colonic macrophages to a proinflammatory phenotype in DOX-induced cardiotoxicity [10]. Here, we supplied butyrate or *P. loescheii* to BMS-1 (10 mg/kg)-treated mice, and found that *P. loescheii* colonization and butyrate supplementation inhibited the BMS-1-induced increase in the number of M1-like (CD68^+^iNOS^+^) cells and decrease in the number of M2-like (CD68^+^CD206^+^) cells in the colonic tissues (Fig. 6A). *P. loescheii* colonization and butyrate supplementation downregulated the mRNA expression of M1 genes (iNOS and CXCL9), but upregulated the expression of M2 genes (CD206 and Arg-1) (Fig. 6B). In addition, we observed similar alterations in the levels of M1-like factors (IL-1β and TNF-α) and M2-like factors (TGF-β and IL-10) in colonic macrophages (Fig. 6C). These data suggested that *P. loescheii* colonization and butyrate supplementation prevent M1-like polarization of colonic macrophages and the production of proinflammatory factors IL-1β and TNF-α in PD-1/PD-L1 inhibitor-related cardiotoxicity.

### Butyrate prevents NF-κB-mediated M1-like polarization and cardiomyocyte apoptosis via PPARα activation

**Fig. 7:**
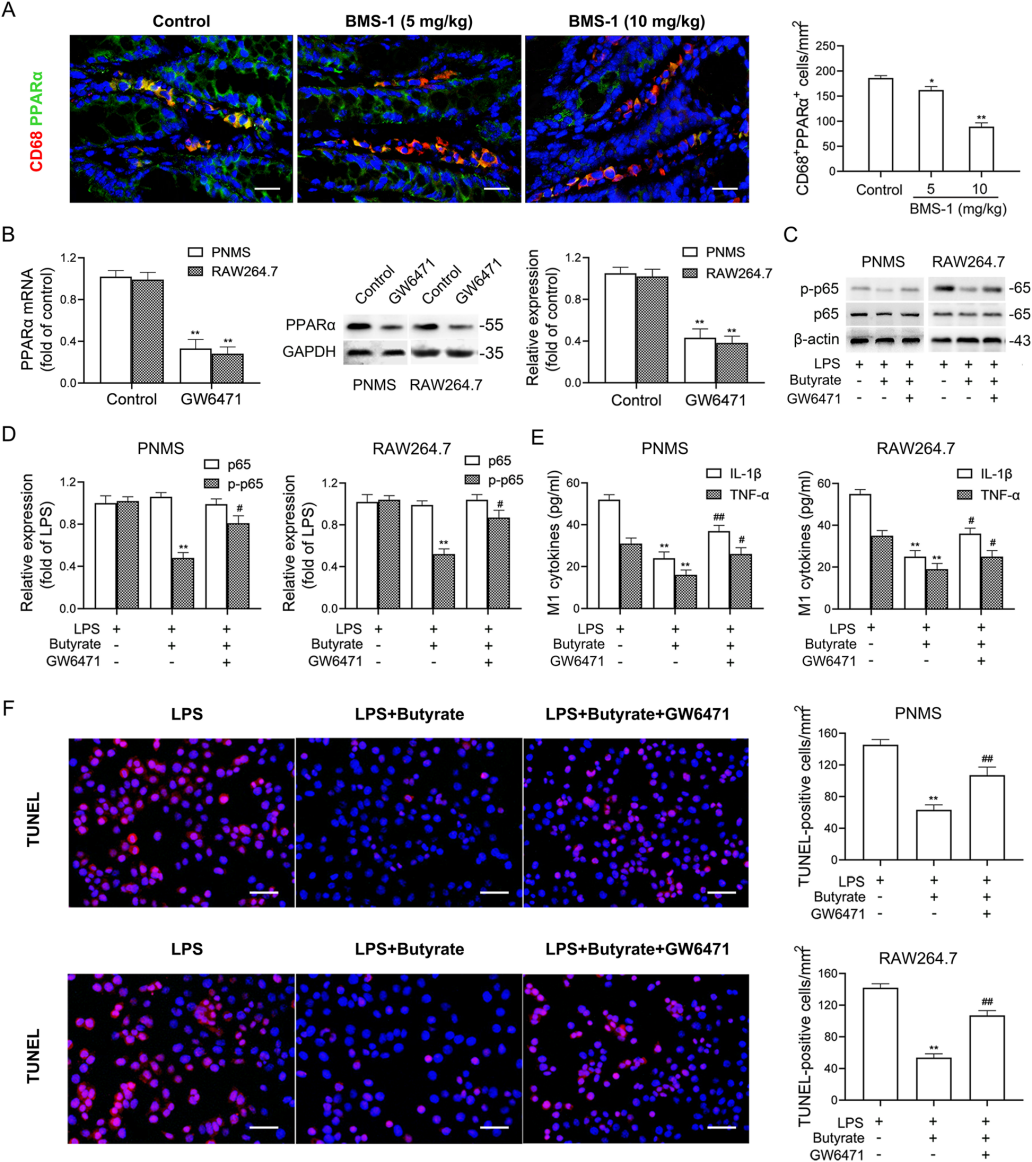
Butyrate prevents NF-κB-mediated M1-like polarization and cardiomyocyte apoptosis via PPARα activation. In the B16F10 melanoma model, the mice were intraperitoneally administered with the PD-1/PD-L1 inhibitor BMS-1 (0, 5 and 10 mg/kg) every 2 days for 6 times (*n* = 10). **A** Immunostaining analysis of the CD68^+^PPARα^+^ cells in colonic tissues. Scale bars, 20 μm. **B** HL-1 cardiomyocytes were co-cultured with the conditioned medium (CM) from peritoneal macrophages (PNMS) and RAW264.7 cells treated with the LPS plus butyrate and GW6471. The efficacy of GW6471 to inhibit PPARα expression was determined by western blot and qPCR (*n* = 5). **C**-**D** Representative western blot images of p65 and p-p65 in PNMS and RAW264.7 cells and corresponding quantification analysis. **E** M1 factors (IL-1β and TNF-α) in PNMS and RAW264.7 cells were determined by ELISA. **F** Representative images of TUNEL assay of HL-1 cardiomyocytes and corresponding quantification analysis. Scale bars, 50 μm. The values are presented as the mean ± standard error of the mean. ^*^P < 0.05, ^**^P < 0.01 vs. control or LPS. ^#^*P* < 0.05, ^##^*P* < 0.01 vs. LPS + butyrate

**Fig. 8:**
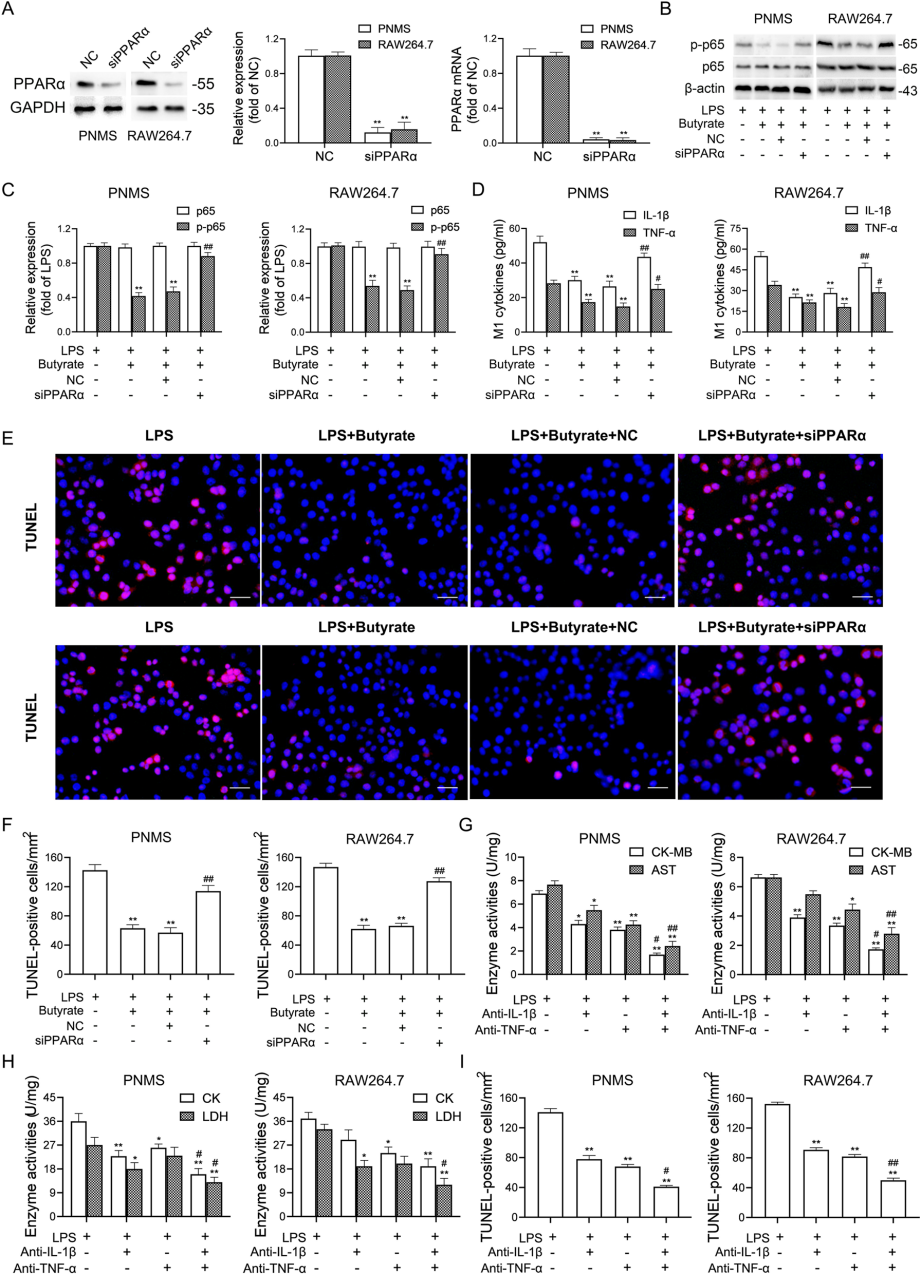
Butyrate prevents NF-κB-mediated M1-like polarization and cardiomyocyte apoptosis via PPARα activation. HL-1 cardiomyocytes were co-cultured with the conditioned medium (CM) from peritoneal macrophages (PNMS) and RAW264.7 cells transfected with PPARα siRNA or negative control siRNA (*n* = 5). **A** The efficacy of PPARα siRNA to silence PPARα was determined by western blot and qPCR. **B**-**C** Representative western blot images of p65 and p-p65 in PNMS and RAW264.7 cells and corresponding quantification analysis. **D** IL-1β and TNF-α in PNMS and RAW264.7 cells were determined by ELISA. **E**-**F** Representative images of TUNEL assay of HL-1 cardiomyocytes and corresponding quantification analysis. Scale bars, 50 μm. **G**-**H** HL-1 cardiomyocytes were co-cultured with the conditioned medium (CM) from PNMS and RAW264.7 cells treated with the LPS plus anti-IL-1β antibody, anti-TNF-α antibody or their combination. The serum levels of creatine kinase-MB (CK-MB), aspartate transaminase (AST), creatine kinase (CK) and lactate dehydrogenase (LDH) were measured. **I** Corresponding quantification analysis of TUNEL assay of HL-1 cardiomyocytes. Scale bars, 50 μm. The values are presented as the mean ± standard error of the mean. ^*^P < 0.05, ^**^P < 0.01 vs. LPS. ^#^*P* < 0.05, ^##^*P* < 0.01 vs. LPS + butyrate or LPS + anti-IL-1β

A previous study showed that PPARα, a ligand of butyrate [29], negatively regulates M1 macrophage polarization by interacting with p65 to abolish its binding to the NF-κB response element [30]. We found that BMS-1 (5 and 10 mg/kg) significantly decreased the expression of PPARα in colonic macrophages (Fig. 7A). To test the role of PPARα in colonic macrophage polarization and thus cardiomyocyte apoptosis, we cocultured HL-1 cardiomyocytes with CM from PNMS or RAW264.7 cells treated with LPS plus butyrate and GW6471 (PPARα antagonist). The efficacy of GW6471 in inhibiting PPARα expression was determined by western blot and qPCR (Fig. 7B). As shown in Fig. 7C-F, butyrate prevented LPS-induced upregulation of p-p65, TNF-α and IL-1β in PNMS and RAW264.7 cells, and decreased the number of TUNEL-positive HL-1 cardiomyocytes. In contrast, the PPARα antagonist GW6471 abolished the protective effect of butyrate. Similar results from the PPARα siRNA silencing experiment confirmed that butyrate prevented LPS-induced M1-like polarization and cardiomyocyte apoptosis in a PPARα-dependent manner (Fig. 8A-F). In addition, we cocultured HL-1 cardiomyocytes with CM from PNMS or RAW264.7 cells treated with LPS plus anti-IL-1β or anti-TNF-α antibody, and found that anti-IL-1β or anti-TNF-α antibody partly abolished LPS-induced cardiotoxicity, as demonstrated by the decreased levels of myocardial enzymes and the number of TUNEL-positive cardiomyocytes. Importantly, a combined block of IL-1β and TNF-α showed better efficacy than a single block (Fig. 8G-I). These data suggested that butyrate prevents NF-κB-mediated M1-like polarization and cardiomyocyte apoptosis via PPARα activation.

### Downregulation of CYP4X1 by a PD-1/PD-L1 inhibitor inactivates PPARα and forms a positive feedback loop in colonic macrophages

**Fig. 9:**
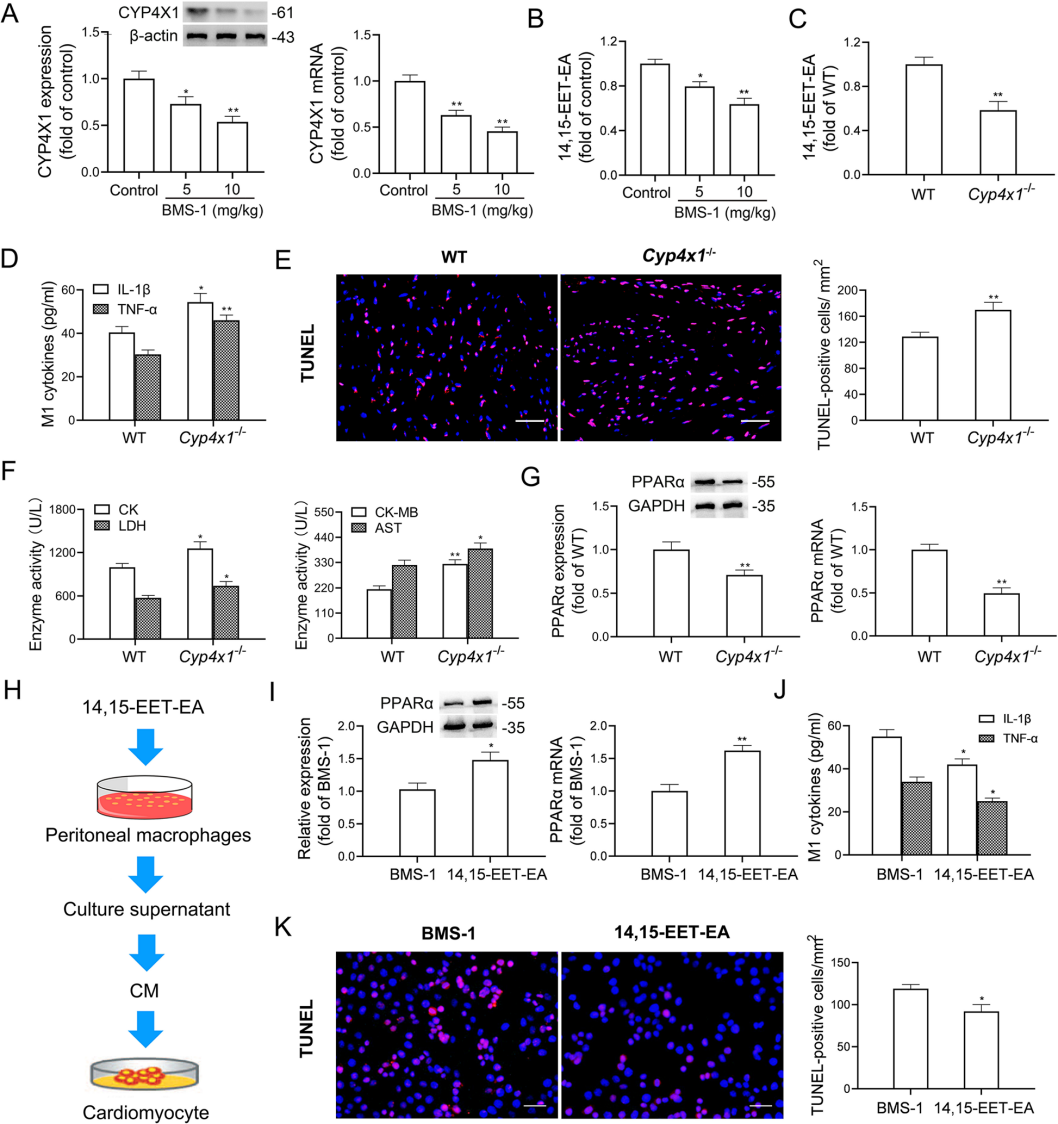
Downregulation of CYP4X1 by a PD-1/PD-L1 inhibitor inactivates PPARα and forms a positive feedback loop in colonic macrophages. In the B16F10 melanoma model, the mice were intraperitoneally administered with the PD-1/PD-L1 inhibitor BMS-1 (0, 5 and 10 mg/kg) every 2 days for 6 times (*n* = 10). **A** CYP4X1 mRNA and protein levels in colonic macrophages. **B** The level of 14,15-EET-EA in colonic macrophages was determined by liquid chromatography tandem-mass spectrometry (LC-MS/MS). **C** In the B16F10 melanoma model, the wild type (WT) and *Cyp4x1* knockout mice (*Cyp4x1*^*−/−*^) mice were intraperitoneally administered with BMS-1 (10 mg/kg) every two days for 6 times (*n* = 10). The production of 14,15-EET-EA in colonic macrophages was determined by LC-MS/MS. **D** M1 factors (IL-1β and TNF-α) in colonic macrophages were determined by ELISA. **E** Representative images of TUNEL assay of cardiac tissues and corresponding quantification analysis. Scale bars, 50 μm. **F** The serum levels of creatine kinase (CK), lactate dehydrogenase (LDH), creatine kinase-MB (CK-MB) and aspartate transaminase (AST). **G** PPARα mRNA and protein levels in colonic macrophages. **H** HL-1 cardiomyocytes were treated with the conditioned medium (CM) from the peritoneal macrophages (PNMS) in the mice treated with PD-1/PD-L1 inhibitor plus 14,15-EET-EA (*n* = 5). **I** PPARα mRNA and protein levels in the PNMS. **J** M1 factors (IL-1β and TNF-α) in the PNMS. **K** Representative images of TUNEL assay of HL-1 cardiomyocytes and corresponding quantification analysis. Scale bars, 50 μm. The values are presented as the mean ± standard error of the mean. ^*^P < 0.05, ^**^P < 0.01 vs. control, WT or BMS-1

Our recent study showed that CYP4X1 inhibition repolarizes TAMs to an M1-like phenotype [15]. Thus, we measured CYP4X1 expression in colonic macrophages. As shown in Fig. 9A, BMS-1 (5 and 10 mg/kg) significantly decreased CYP4X1 expression in colonic macrophages. Moreover, the catalytic production of 14,15-EET-EA derived from CYP4X1 was significantly decreased in colonic macrophages from the mice treated with BMS-1 (5 and 10 mg/kg) compared with the control (Fig. 9B). Next, the WT and *Cyp4x1*^*−/−*^ mice were treated with BMS-1 (10 mg/kg). As shown in Fig. 9C and D, a decrease in 14,15-EET-EA production and an increase in TNF-α and IL-1β secretion were observed in colonic macrophages from *Cyp4x1*^−/−^ mice compared with those from WT mice. In addition, we observed more TUNEL-positive cardiomyocytes and higher serum levels of myocardial enzymes (CK, LDH, CK-MB and AST) in *Cyp4x1*^*−/−*^ mice (Fig. 9E and F). A previous study showed that PPARα activates human CYP4X1 gene transcription [31]. We found that PPARα siRNA downregulated CYP4X1 (Fig. S2), and in turn, *Cyp4x1* knockout downregulated PPARα mRNA and protein expression in colonic macrophages (Fig. 9G). Exogenous supplementation with 14,15-EET-EA significantly attenuated the decrease in PPARα expression and the increase in IL-1β and TNF-α production in PNMS, and thus reversed the cardiomyocyte apoptosis induced by BMS-1 (Fig. 9H-K). These data suggested that downregulation of CYP4X1 by a PD-1/PD-L1 inhibitor inactivates PPARα and forms a positive feedback loop in colonic macrophages.

## Discussion

**Fig. 10:**
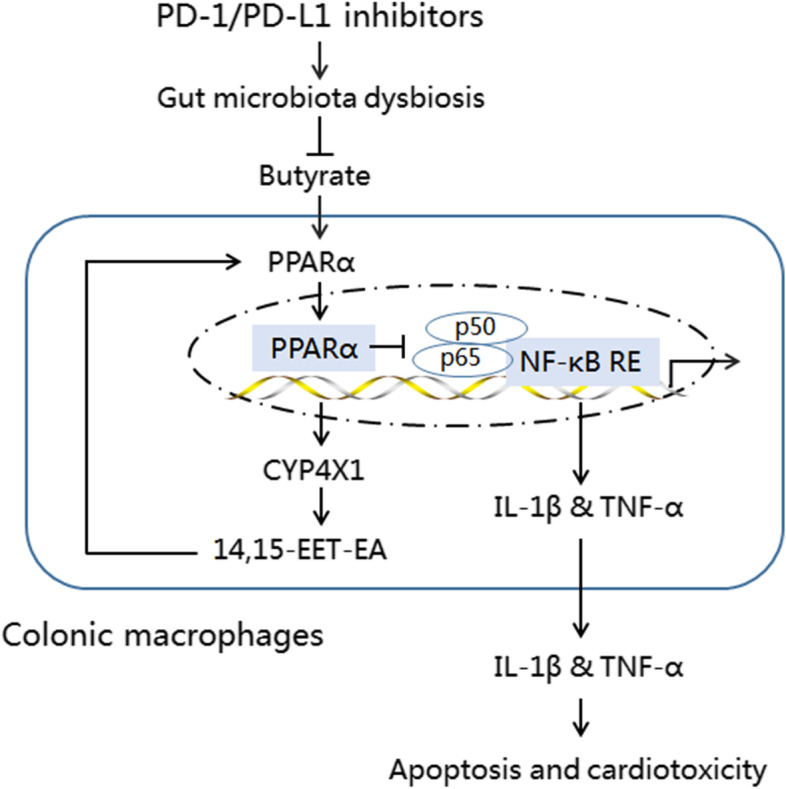
Prevotellaceae produces butyrate to alleviate PD-1/PD-L1 inhibitor-related cardiotoxicity through the upregulation of PPARα-CYP4X1 axis in colonic macrophages. 14,15-EET-EA, 14,15-epoxyeicosatetraenoic acid ethanolamide; CYP4X1, cytochrome P450 4X1; IL-1β, interleukin 1β; NF-κB, nuclear transcription factor kappa B; PPARα, peroxisome proliferator-activated receptor α; RE, response element; TNF-α, tumor necrosis factor-α

In the present study, we provide the first direct evidence that PD-1/PD-L1 inhibitors induce cardiomyocyte apoptosis and cardiotoxicity in a gut microbiota-dependent manner, and that *P. loescheii* colonization and butyrate supplementation play a protective role in PD-1/PD-L1 inhibitor-related cardiotoxicity. In addition, we found that PD-1/PD-L1 inhibitor-induced gut microbiota dysbiosis upregulated proinflammatory factors IL-1β and TNF-α in colonic macrophages. To our knowledge, this is the first study to demonstrate the crosstalk between gut microbiota and colonic macrophages in PD-1/PD-L1 inhibitor-related cardiotoxicity. Importantly, we demonstrated for the first time that inhibition of the PPARα and CYP4X1–14,15EET-EA positive feedback loop promoted M1-like polarization of colonic macrophages and the production of TNF-α and IL-1β. Our results identify a novel mechanism of action of PD-1/PD-L1 inhibitor-related cardiotoxicity (Fig. 10), and manipulating the gut microbiota *P. loescheii* and its metabolite butyrate to modulate the PPARα-CYP4X1 axis in colonic macrophages could reduce the risk of cardiotoxicity following anti-PD-1/PD-L1 treatment*.*

Gut microbiota, including *Prevotellaceae*, *Rikenellaceae* and *Ruminococcaceae* and their metabolites SCFAs play important roles in intestinal barrier integrity and intestinal homeostasis [32]. *Rikenellaceae*, which belongs to the Bacteroidetes phylum, is correlated with resistance to CTLA-4 inhibitor-related colitis [33], and a reduction in butyrate-producing taxa promotes systemic inflammation and atherosclerosis [34]. In contrast, the expansion of Proteobacteria, specifically *Escherichia-Shigella*, has a positive correlation with immunosuppressive drug-induced gastrointestinal toxicity [35]. Here, we observed gut microbiota dysbiosis characterized by depletion of *Prevotellaceae* and *Rikenellaceae* genus, enrichment of *Escherichia-Shigella* and *Ruminococcaceae* genus and low microbial butyrate production in PD-1/PD-L1 inhibitor-induced cardiotoxicity in mice. Importantly, we first demonstrated that a PD-1/PD-L1 inhibitor induced cardiomyocyte apoptosis and cardiotoxicity, at least partly through *P. loescheii* depletion and low butyrate production in gut microbiota. We concluded that in addition to *P. loescheii* depletion and low butyrate production, *Escherichia-Shigella* enrichment and high LPS production might also contribute to PD-1/PD-L1-related cardiotoxicity, and additional experiments are being carried out to investigate this possibility. A previous study showed that enrichment of *Prevotellaceae*, *Ruminococcaceae*, and *Lachnospiraceae* confers a preferred response to anti-PD-1/PD-L1 treatment [36]. Here we found that *P. loescheii* recolonization and butyrate supplementation decreased the tumor weight and luciferase intensity (Fig. S3), suggesting that *P. loescheii* improves the efficacy in addition to reducing the cardiotoxicity of anti-PD-1/PD-L1 treatment. Our studies were conducted in mice, and whether *P. loescheii* and butyrate could influence the efficacy and cardiotoxicity of PD-1/PD-L1 inhibitors in humans remains to be determined. Previous studies showed that the enrichment of *Prevotellaceae*, *Rikenellaceae* and *Bacteroides* in the gut microbiota contributes to acute myocardial ischemia in rats [37], and the abundance of *Ruminococcaceae* bacteria is negatively correlated with isoproterenol-induced arterial stiffness [38]. This discrepancy may be due to the differences in the pathological conditions (apoptosis versus arterial stiffness), exogenous toxin (PD-1/PD-L1 inhibitor versus isoproterenol) and the animal models (C57BL/6 mice versus Wistar rats), and future efforts are warranted to clarify these possibilities.

Colonic macrophages provides an active target for potential novel therapies in maintaining homeostasis and resisting inflammation [39]. Macrophages in peritoneal exudate are polarized to an M1 phenotype in PD-1-deficient mice [40]. Our recent study showed that colonic macrophages are polarized to an M1-like phenotype in DOX-induced cardiotoxicity, and reprogramming colonic macrophages away from the M1-like phenotype attenuates DOX-induced acute cardiotoxicity [10]. Here, we demonstrated that PD-1/PD-L1 inhibitor-induced gut microbiota dysbiosis and low butyrate production upregulated proinflammatory factors IL-1β and TNF-α in colonic macrophages, and that depleting colonic macrophages or a combination of IL-1β and TNF-α blockade significantly attenuated PD-1/PD-L1 inhibitor-related cardiotoxicity. These data suggest that M1-like colonic macrophages and their proinflammatory factors IL-1β and TNF-α contribute to PD-1/PD-L1 inhibitor-related cardiotoxicity. Given that gut microbiota dysbiosis mediates PD-1/PD-L1 inhibitor-related cardiotoxicity through multiple proinflammatory factors, including IL-1β and TNF-α, novel targeted therapies founded on gut microbiota not any single cytokine, may represent a potential strategy for the prevention of PD-1/PD-L1 inhibitor-related cardiotoxicity.

PPARα, a ligand-activated nuclear receptor, is involved not only in lipid metabolism but also in inflammatory reactions [41]. PPARα inhibits the expression of proinflammatory factors and macrophage M1-like polarization mainly by NF-κB downregulation [30]. Butyrate, one of the natural agonists of PPARα [29], is decreased in M1-like colonic macrophages [10]. In this study, we demonstrated that downregulation of PPARα in colonic macrophages by low butyrate production attenuated its inhibitory effects on NF-κB signaling, and thus upregulated the proinflammatory factors IL-1β and TNF-α. A previous study showed that PPARα activation upregulates human CYP4X1 gene transcription [31], and CYP4X1-derived cannabinoid metabolites further activated PPARα [42]. Consistently, we found that *Cyp4x1* knockout inhibited PPARα expression in colonic macrophages, but exogenous supplementation with 14,15-EET-EA promoted PPARα expression in the PNMS, thus attenuating PD-1/PD-L1 inhibitor-induced the upregulation of IL-1β and TNF-α. These data suggest that inactivation of the PPARα and CYP4X1–14,15-EET-EA positive feedback loop in colonic macrophages upregulates proinflammatory factors IL-1β and TNF-α, and thereby amplifies PD-1/PD-L1 inhibitor-induced cardiotoxicity.

Although there were important discoveries revealed by this study, some limitations still exist. This study mainly focused on PD-1/PD-L1 inhibitor-associated myocarditis, and other forms of ICI-related cardiotoxicity need to be further investigated. In addition, the mechanism of PD-1/PD-L1 inhibitor-related cardiotoxicity was only investigated in BMS-1-treated mice. Additional experiments with larger sample sizes in anti-PD-1/PD-L1 antibody-treated mice and humans will be performed to validate these results.

## Conclusions

Gut microbiota dysbiosis-induced low butyrate production contributes to PD-1/PD-L1 inhibitor-related cardiotoxicity through downregulation of PPARα-CYP4X1 axis in colonic macrophages. Importantly, our study identified the crosstalk between the gut microbiota and colonic macrophages in PD-1/PD-L1 inhibitor-related cardiotoxicity. Our findings propose a novel mechanism of action of PD-1/PD-L1 inhibitor-related cardiotoxicity, and may provide a notable target for making immunotherapy safer and more effective by manipulating the gut microbiota and its metabolite butyrate.

## Supplementary Information

**Additional file 1.**

## Data Availability

The datasets generated or analyzed during the current study are available on reasonable request.

## Abbreviations

14,15-EET-EA: 14,15-epoxyeicosatetraenoic acid ethanolamide
ABPAS: Alcian blue-periodic acid-Schiff
Abx: broad-spectrum antibiotic
AST: aminotransferase
Bax: B-cell lymphoma protein 2-associated X
Bcl-2: B-cell lymphoma-2
BNP: brain natriuretic peptide
CK: creatine kinase
CK-MB: creatine kinase-MB
CM: conditioned medium
CYP4X1: cytochrome P450 4X1
ELISA: enzyme-linked immunosorbent assay
HAX-1: HS-associated protein X-1
HE: Hematoxylin and Eosin
HPLC: high performance liquid chromatography
IL-1β: interleukin 1β
irAEs: immune-related adverse events
LC-MS/MS: liquid chromatographic-tandem mass spectrometry
LDH: lactate dehydrogenase
LEfSe: linear discriminant analysis effect size
NF-κB: nuclear transcription factor kappa B
OTUs: Operational Taxonomic Units
PBS: phosphate buffer saline
PCNA: proliferating cell nuclear antigen
PCoA: principal coordinates analysis
PD-1: programmed cell death protein 1
PD-L1: programmed death ligand 1
PNMS: peritoneal macrophages
PPARα: peroxisome proliferator-activated receptor α
*P. loescheii*: *Prevotella loescheii*
RE: response element
SCFAs: short-chain fatty acids
SEM: standard error of the mean
TNF-α: tumor necrosis factor-α
TUNEL: terminal deoxynucleotidyl transferase dUTP nick end labeling
