# Supplementary material for: Prevotellaceae produces butyrate to alleviate PD-1/PD-L1 inhibitor-related cardiotoxicity via PPARα-CYP4X1 axis in colonic macrophages
Source: J Exp Clin Cancer Res. 2022 Jan 3;41:1. doi: 10.1186/s13046-021-02201-4 (PMC8722009; doi:10.1186/s13046-021-02201-4)
Supplement: Supplementary file 1 — Additional file 1. [file 13046_2021_2201_MOESM1_ESM.zip › Supplemental Material.docx]

**SUPPLEMENTARY MATERIAL**

**Prevotellaceae produces butyrate to alleviate PD-1/PD-L1 inhibitor-related cardiotoxicity *via* PPARα-CYP4X1 axis in colonic macrophages**

Yaxin Chen^a#^, Yanzhuo Liu^b#^, Yang Wang*^c^*, Xuewei Chen*^d^*, Chenlong Wang*^e^*, Xuehan Chen^c^, Xi Yuan^c^, Lilong Liu^c^, Jing Yang^c^*, Xiaoyang Zhou^a^*

**Figure S1** **The rarefaction curves of the bacterial community.** In the B16F10 melanoma model, the mice were intraperitoneally administered with PD-1/PD-L1 inhibitor BMS-1 (0, 5 and 10 mg/kg) every two days for 6 times (*n* = 4). The values are presented as the mean ± standard error of the mean. ^*^*P* < 0.05, ^**^*P* < 0.01 *vs*. control.

**Figure S2 The downregulation of PPARα inhibits CYP4X1 gene transcription.** RAW264.7 cells were treated with PPARα siRNA (*n* = 5). (A) PPARα-binding elements on the promoters of CYP4X1 genes were predicted by JASPAR. (B-C) The relative expression of CYP4X1 in RAW264.7 cells treated with the PPARα siRNA was determined by western blot and qPCR. The values are presented as the mean ± standard error of the mean. ^*^*P* < 0.05, ^**^*P* < 0.01 *vs*. negative control (NC).

**Figure S3** ***P. loescheii* recolonization and butyrate supplementation improve the efficacy of PD-1/PD-L1 inhibitor.** In the B16F10 melanoma model, the C57BL/6 mice were orally administrated with *Prevotellaceae* *loescheii* (*P. loescheii*) (1×10^8^ CFU/mouse/every 2 days) or sodium butyrate (1 g/kg) for 1 week during BMS-1 (10 mg/kg) treatment (*n* = 10). (A) Tumor weight of the mice. (B) Representative bioluminescence images of mice bearing tumors on day 15 after implantation. Signal intensity was measured as photon flux (photons/second) and coded to a color scale. The values are presented as the mean ± standard error of the mean. ^*^*P* < 0.05, ^**^*P* < 0.01 *vs*. BMS-1.

**SUPPLEMENTARY METHODS**

**Histopathological examination of cardiac and colonic tissues**

The cardiac tissue sections of the mice from BMS-1 (5 and 10 mg/kg) and control groups were stained with Hematoxylin and Eosin (HE) or Masson’s Trichome. The slides were evaluated by two pathologists who were masked to the treatment protocol and results. For quantification, inflammatory foci number and the percentage of the area of fibrosis to that of the entire tissue specimen were calculated using Image J software (1.41v, US National Institutes of Health, USA), and 10 randomly fields at a magnification of 200× were chosen for each sample.

The colonic tissue sections of the mice from BMS-1 (5 and 10 mg/kg) and control groups were stained with Alcian blue-periodic acid-Schiff (ABPAS) and HE. Villus height were determined in 10 different sections at a magnification of 200×. To count goblet cells, the total number of PAS-positive cells was determined in 10 longitudinally sectioned crypts of villi of colonic tissues per section.

**TUNEL assay**

The apoptotic cell numbers in cardiac tissues from BMS-1 (5 and 10 mg/kg) and vehicle-treated mice was evaluated by the terminal deoxynucleotidyl transferase dUTP nick end labeling (TUNEL) kit (C1090, Beyotime Biotechnology, Shanghai, China) as previously described [1]. Immunofluorescence procedures for detecting apoptotic cardiomyocytes were performed according to the manufacturer’s instructions by using an Olympus BX51 microscope (Olympus, Tokyo, Japan). For each slide, 10 fields were randomly chosen, and using a defined rectangular field area (40 × objective). The index of apoptosis (number of positively stained apoptotic myocytes/rectangular field area) was determined using Image J software (1.41v, US National Institutes of Health, USA).

**Detection of serum biochemical indexes**

The serum levels of creatine kinase (CK) (A032), lactate dehydrogenase (LDH) (A020-2), creatine kinase-MB (CK-MB) (E006) and aminotransferase (AST) (C010-2) were detected according to the instructions of kits (Nanjing Jiancheng Bioengineering Institute, Nanjing, China).

**Quantification of 14,15-EET-EA**

Liquid chromatography tandem-mass spectrometry (LC-MS/MS) was used to analyze 14,15-epoxyeicosatetraenoic acid ethanolamide (14,15-EET-EA) as our previous description [2]. Briefly, the samples were injected onto a Hypersil ODS column (5 mm, 4.6 × 100 mm; Thermo Fisher Scientific, Waltham, MA) that had been equilibrated with 75% solvent B (0.1% acetic acid in methanol) and 25% solvent A (0.1% acetic acid in water). The metabolites were resolved using the following gradient: 0-5 minutes, 75% B; 5-20 minutes, 75%-100% B; 20-25 minutes, 100% B; 25-26 minutes, 100%-75% B; and 26-30 minutes, 75% B. The flow rate was 0.3 ml/min. The column effluent was directed into the Agilent LSD ion trap mass spectrometer 1100 (Agilent Technologies, Palo Alto, CA) using negative electrospray ionization-MS/MS, and the peaks eluting with a mass/charge ratio (m/z) of 364 (14,15-EET-EA) were isolated and monitored.

**Immunofluorescence**

The colonic tissues were labeled with the antibody against PCNA (1:100), CD68 (1:100) plus iNOS (1:100), CD206 (1:500), PPARα (1:200) or CYP4X1 (1:100) overnight at 4 °C. After washing, the sections were incubated with a mixture of Alexa Fluor 488- and Alexa Fluor 594-conjugated secondary antibodies for 1 h. Nuclei were counterstained with 40, 6-diamidino-2-phenylindole (DAPI). Each section was observed by using an Olympus BX51 microscope (Olympus, Tokyo, Japan). For quantification, the index of positively stained cells (the number of PCNA^+^, CD68^+^ iNOS^+^, CD68^+^ CD206^+^, CD68^+^ PPARα^+^ or CD68^+^ CYP4X1^+^ cells/rectangular field area) was determined by 10 randomly chosen fields at 400× magnification for each sample.

**Cytokine measurement**

The production of M1 factors (TNF-α and IL-1β) and M2 factors (IL-10 and TGF-β) in colonic macrophages from BMS-1 (5 and 10 mg/kg), *Cyp4x1^-/-^*, *Prevotellaceae* *loescheii*, butyrate, GW6471, 14,15-EET-EA and control groups were measured by using enzyme-linked immunosorbent assay (ELISA) kits from R&D Systems ( MTA00B, MLB00C, M1000B, DB100B, Minneapolis, MN, United States) according to the manufacturer's instruction.

**Western blot analysis**

Protein in cardiac and colonic tissues were extracted from BMS-1 (5 and 10 mg/kg) and control groups. Based on the concentration determined by bicinchoninic acid method, equal amounts of protein extracts were subjected to electrophoresis in SDS-polyacrylamide gels and transferred to polyvinylidene fluoride membranes. The membrane was blocked with 5% nonfat milk and probed with primary antibodies against cleaved caspase 9 (1:1000), cleaved caspase 3 (1:1000), Bax (1:1000), Bcl-2 (1:1000), HAX-1 (1:200), Claudin-1(1:1000), Occludin (1:1000), ZO-1(1:1000), PPARα (1:1000), CYP4X1 (1:500), p65 (1:1000), phosphor-p65 (1:1000) and β-actin (1:1000). The antibodies were detected using peroxidase-conjugated anti-rabbit and anti-mouse immunoglobulin G (Proteintech, Chicago, Illinois, USA), and blots were detected using the ECL system (Thermo Fisher Scientific, Waltham, MA, USA). The relative expression was normalized to the expression of β-actin.

**qPCR analysis**

Total RNA was extracted from the isolated colonic macrophage using Trizol (15596018, Invitrogen, Carlsbad, CA, United States) according to the manufacturer’s instruction. RNA concentration and purity were estimated from the optical density at 260 and 280 nm, respectively. Total RNA was subjected to cDNA synthesis using PrimeScript™ RT reagent Kit with gDNA Eraser (RR047A, Takara, Dalian, Liaoning, China). PCR amplification was performed with the CFX96 Real Time System (Bio-Rad, Hercules, CA, United States), using SYBR Premix Ex Taq TM (2×; Takara, Japan). The primer pairs of BNP, iNOS, CXCL9, arginase-1, CD206, PPARα, CYP4X1, β-actin and *Prevotellaceae* *loescheii* (*P. loescheii*) were designed and synthesized according to previous studies [2-6], and the sequences of the primer were listed in Table 1. Relative quantification was performed using the 2^−△△Ct^ method.

← Table 1

← Figure 1

← Figure 1

← Figure 1

← Figure 1

**Table 1 Primer pairs used in the present study.**

| Primers | Forward primer (5’-3’) | Reverse primer (5’-3’) |
| --- | --- | --- |
| iNOS | GTTCTCAGCCCAACAATACAAGA | GTGGACGGGTCGATGTCAC |
| BNP | AATTCAAGATGCAGAAGCTG | GAATTTTGAGGTCTCTGCTG |
| CXCL8 | CCGAGGCACGTCCACTTACA | TCTAGGTTTGATCCCGTTC |
| CD206 | CAAGGAAGGTTGGCATTTGT | CCTTTCAGTCCTTTGCAAG |
| Arginase-1 | CTCCAAGCCAAAGTCCTTAGAG | AGGAGCTGTCATTAGGGACATC |
| PPARα | TCATCAAGAAGACGGAGTCG | CGGTTACCTACAGCTCAGAC |
| CYP4X1 | GAAGATATTTCTGAGCAGAA | TCGATGGTTGTTTCCTG |
| β-actin | TGACAGGATGCAGAAGGAGA | TAGAGCCACCAATCCACACA |
| *P. loeschei*i | CACCAAGGCGACGATCA | GGATAACGCCYGGACCT |

**REFERENCES**

1. Liu X, Liu Y, Chen X, Wang C, Chen X, Liu W, et al. Multi-walled carbon nanotubes exacerbate doxorubicin-induced cardiotoxicity by altering gut microbiota and pulmonary and colonic macrophage phenotype in mice. Toxicology. 2020;435:152410.

2. Wang C, Li Y, Chen H, Huang K, Liu X, Qiu M, et al. CYP4X1 inhibition by flavonoid CH625 normalizes glioma vasculature through reprogramming TAMs via CB2 and EGFR-STAT3 axis. J Pharmacol Exp Ther. 2018;365(1):72-83.

3. Cao Z, Zhang T, Xu C, Jia Y, Wang T, Zhu B. AIN-93 diet as an alternative model to lieber-decarli diet for alcoholic cardiomyopathy. Alcoholism, clinical and experimental research. 2019;43(7):1452-61.

4. Huang K, Liu Y, Tang H, Qiu M, Li C, Duan C, et al. Glabridin prevents doxorubicin-induced cardiotoxicity through gut microbiota modulation and colonic macrophage polarization in mice. Front Pharmacol. 2019;10:107.

5. Chung KW, Lee EK, Lee MK, Oh GT, Yu BP, Chung HY. Impairment of PPARalpha and the fatty acid oxidation pathway aggravates renal fibrosis during aging. J Am Soc Nephrol. 2018;29(4):1223-37.

6. Yoshida A, Tachibana M, Ansai T, Takehara T. Multiplex polymerase chain reaction assay for simultaneous detection of black-pigmented Prevotella species in oral specimens. Oral Microbiol Immunol. 2005;20(1):43-6.
