# Supplementary figures and images for: Prevotellaceae produces butyrate to alleviate PD-1/PD-L1 inhibitor-related cardiotoxicity via PPARα-CYP4X1 axis in colonic macrophages
Source: J Exp Clin Cancer Res. 2022 Jan 3;41:1. doi: 10.1186/s13046-021-02201-4 (PMC8722009; doi:10.1186/s13046-021-02201-4)

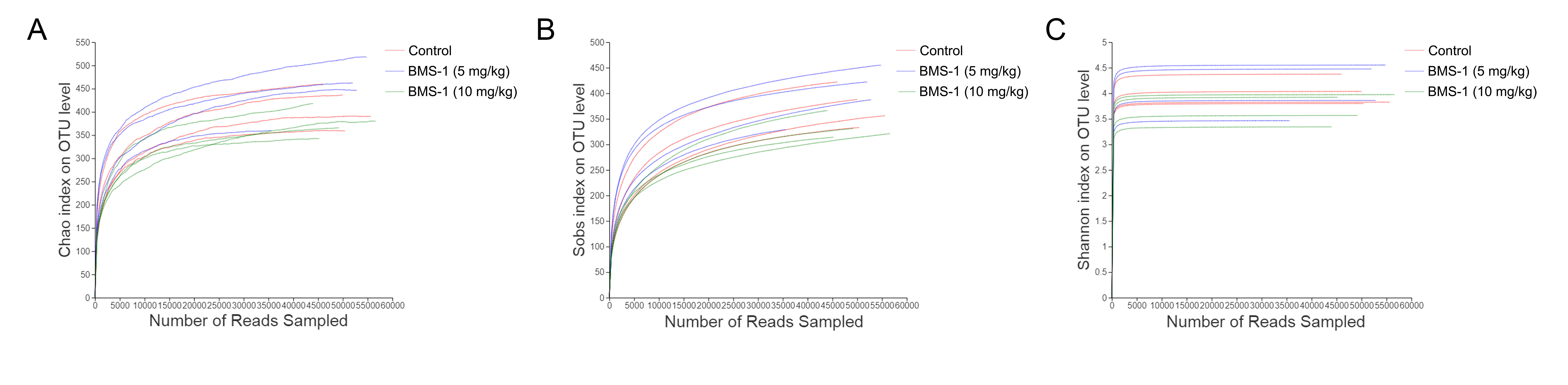

Supplement: Supplementary file 1 — Additional file 1. [file 13046_2021_2201_MOESM1_ESM.zip › S1.jpg]

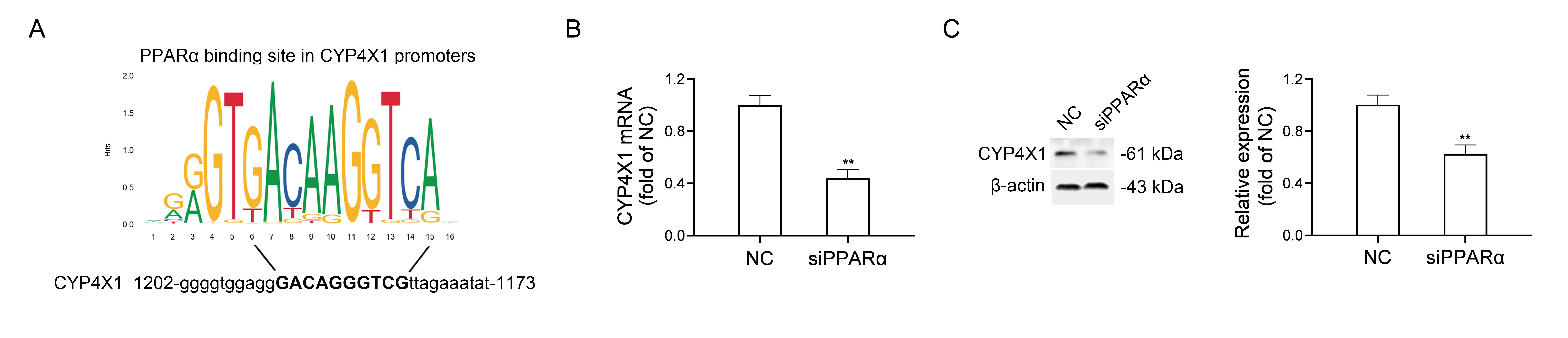

Supplement: Supplementary file 1 — Additional file 1. [file 13046_2021_2201_MOESM1_ESM.zip › S2.jpg]

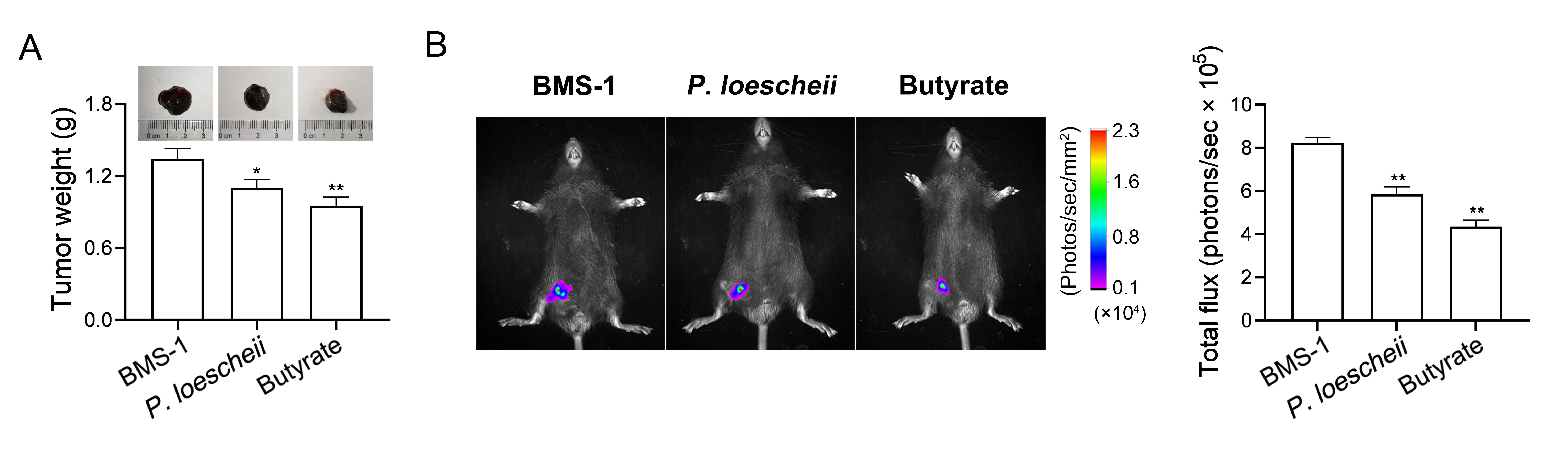

Supplement: Supplementary file 1 — Additional file 1. [file 13046_2021_2201_MOESM1_ESM.zip › S3.jpg]
